# The Interaction Mechanism Between Herpes Simplex Virus 1 Glycoprotein D and Host Antiviral Protein Viperin

**DOI:** 10.3389/fimmu.2019.02810

**Published:** 2019-12-11

**Authors:** Meili Li, Zongmin Liao, Zuo Xu, Xingmei Zou, Yuanfang Wang, Hao Peng, Yiwen Li, Xiaowen Ou, Yangxi Deng, Yingjie Guo, Weidong Gan, Tao Peng, Daixiong Chen, Mingsheng Cai

**Affiliations:** ^1^Guangdong Provincial Key Laboratory of Allergy and Clinical Immunology, Second Affiliated Hospital of Guangzhou Medical University, Guangzhou, China; ^2^Department of Pathogenic Biology and Immunology, School of Basic Medical Science, Sino-French Hoffmann Institute, Guangzhou Medical University, Guangzhou, China; ^3^Department of Scientific Research and Education, Yuebei People's Hospital, Shaoguan, China; ^4^State Key Laboratory of Respiratory Diseases, School of Basic Medical Science, Sino-French Hoffmann Institute, Guangzhou Medical University, Guangzhou, China; ^5^South China Vaccine Corporation Limited, Guangzhou, China

**Keywords:** herpes simplex virus 1, viperin, gD, IFN-β, NF-κB

## Abstract

Viperin is an interferon-inducible protein that responsible for a variety of antiviral responses to different viruses. Our previous study has shown that the ribonuclease UL41 of herpes simplex virus 1 (HSV-1) can degrade the mRNA of viperin to promote HSV-1 replication. However, it is not clear whether other HSV-1 encoded proteins can regulate the function of viperin. Here, one novel viperin associated protein, glycoprotein D (gD), was identified. To verify the interaction between gD and viperin, gD and viperin expression plasmids were firstly co-transfected into COS-7 cells, and fluorescence microscope showed they co-localized at the perinuclear region, then this potential interaction was confirmed by co-immunoprecipitation (Co-IP) assays. Moreover, confocal microscopy demonstrated that gD and viperin co-localized at the Golgi body and lipid droplets. Furthermore, dual-luciferase reporter and Co-IP assays showed gD and viperin interaction leaded to the increase of IRF7-mediated IFN-β expression through promoting viperin and IRAK1 interaction and facilitating K63-linked IRAK1 polyubiquitination. Nevertheless, gD inhibited TRAF6-induced NF-κB activity by decreasing the interaction of viperin and TRAF6. In addition, gD restrained viperin-mediated interaction between IRAK1 and TRAF6. Eventually, gD and viperin interaction was corroborated to significantly inhibit the proliferation of HSV-1. Taken together, this study would open up new avenues toward delineating the function and physiological significance of gD and viperin during HSV-1 replication cycle.

## Introduction

Herpes simplex virus 1 (HSV-1), a widespread human pathogen that can cause lytic infection in mucosal epithelial cells and life-long latent infection in neurons, is a nuclear-replicating DNA virus with a genome encoding ~80 different proteins, among which at least 44 proteins are the structural components of the virions. According to their known or putative localizations in the virions, the proteins can be classified into five groups, namely envelope, tegument, capsid, unclassified, and non-structural proteins (1, 2).

HSV-1 glycoproteins are found in the virion envelope as well as membrane of the infected cell, and gD is a multifunctional protein that can interact with three cellular receptors for entry (3), including nectins (nectin 1 and 2) (4, 5), a modified heparin sulfate (6) and herpesvirus entry mediator (HVEM, also named herpesvirus entry protein A) (7), hence it defines the viral tropism. Once binding to the receptor, an ensuing change in gD conformation exposes to profusion domains, which enables fusogenic glycoprotein gB, gH, and gL to complete fusion of the envelope with the plasma membrane (8). Therefore, binding of gD to a cell surface receptor is an essential step of virus entry (8, 9). gD also plays a key role in multiple events during HSV-1 infection, including cell-to-cell spread and virus-induced syncytia formation. However, packaging of gD into virions is almost completely blocked in the absence of tegument protein UL16 (10).

It is well-known that innate immune response is the first line for host defense. When viral infection, virus can activate the host innate antiviral response and result in the expression of series cellular protective genes, e.g., proinflammatory cytokines and type I interferon (IFN-I, including IFN-α, and IFN-β), which then induces a subset of interferon-stimulated genes (ISGs) to reinforce IFN-I signaling and prime cells with enhanced antiviral activity to inhibit viral replication (11, 12).

Viperin is an evolutionarily conserved iron-sulfur (Fe-S) cluster-binding protein (13–15), which can be induced in various cell types by distinct stimuli of IFN-I and IFN-II, viral DNA, dsRNA, polyI:C, LPS, and by infection with diverse viruses, such as human cytomegalovirus (HCMV) (16), pseudorabies virus (17), Japanese encephalitis virus (18), West Nile virus (19), hepatitis C virus (HCV) (15), Chikungunya virus (20), rhinovirus (21), yellow fever virus (22), lymphocytic choriomeningitis virus (23), and dengue virus (DENV) (24). Nevertheless, viperin shows antiviral ability to many types of viruses. For example, viperin can reduce cholesterol/sphingomyelin on the membranes that are the main components of lipid rafts, which are essential for the entry, assembly, and budding of rabies virus in RAW264.7 cells (25). Viperin also can inhibit the release of influenza A virus (IAV) by down-regulating cholesterol synthesis and perturbing lipid rafts, which are required for the stability and infectivity of IAV (26, 27). In addition, viperin can associate with some host and viral proteins, such as mitochondrial antiviral signaling protein (MAVS) (28), signal mediators interleukin-1 receptor-associated kinase 1 (IRAK1), TNF receptor-associated factor 6 (TRAF6) (29), DENV-2 NS3 (30), HCV NS5A (15), and HCMV vMIA (13), and its function is therefore regulated.

Since IFN-I and nuclear factor B (NF-κB) play key roles in regulating the antiviral response (31), HSV-1 has evolved multiple strategies to escape these two innate systems (11, 32). Specifically, US3 protein kinase inhibits the IFN-β-signaling pathway by interacting with and hyperphosphorylating IFN regulatory factors 3 (IRF3) (33), UL36 ubiquitin specific protease deubiquitinates TRAF3 and then blocks IFN-β production (34), VP16 abrogates the interferon antiviral response by suppressing NF-κB and preventing IRF3 to recruit its co-activator, CREB binding protein (35). Our previous study has demonstrated that the ribonuclease UL41 can degrade the mRNA of viperin to restrain its antiviral function (36). However, it is still not clear whether other HSV-1 encoded proteins can interact with viperin, and what is the effect or mechanism of their interaction? Therefore, given viperin plays a very important role in the regulation of host antiviral response, a screening of fluorescence microscope was firstly carried out to find which HSV-1 protein can co-localize with viperin or alter its normal subcellular localization, then their interaction was tested by co-immunoprecipitation (Co-IP) assays, and other experiments such as confocal microscopy, dual-luciferase reporter (DLR) assays and real-time quantitative PCR (RT-qPCR), were performed to explore how this interaction regulates the signaling pathways of IFN-β and NF-κB in the host innate immune system.

## Materials and Methods

### Cells

COS-7 and HEK293T cells were cultured at 37°C in Dulbecco's modified MEM (DMEM. Gibco-BRL) supplemented with 10% heat inactivated fetal bovine serum (FBS, Gibco-BRL).

### Antibodies

Mouse anti-Flag, anti-Myc, anti-hemagglutinin (HA), and anti-β-actin monoclonal antibodies (mAbs) were purchased from ABmart. Rabbit anti-Flag polyclonal antibody (pAb) was purchased from Proteintech. Mouse non-specific control IgG antibody was purchased from eBioscience Inc. Rabbit anti-gD pAb was gifted by Dr. Roselyn J. Eisenberg (School of Veterinary Medicine, University of Pennsylvania), and mouse anti-gD mAb was purchased from Santa Cruz Biotechnology.

### Plasmids Construction

The ORF of viperin was amplified by PCR using pViperin-Flag expression plasmid (provided by Dr. Yi-Ling Lin, Genomics Research Center, Academia Sinica, Taiwan) (18) as the template, which was then cloned into pEGFP-N1 (Clontech) to yield pEGFP-viperin. The US6 ORF of HSV-1 (F strain) glycoprotein D (gD) was also amplified from HSV-1 DNA pYEbac102 (37, 38), with forward primer 5′-AGG AAT TCA TGG GGG GGG CTG CCG CCA GG-3′ and reverse primer 5′-CGG GAT CCT TGT AAA ACA AGG GCT GGT G-3′. The purified PCR product was digested with *Eco*RI and *Bam*HI and then inserted into the corresponding digested pEYFP-N1 (Clontech) to yield plasmid pgD-EYFP, as described previously (39–43). Reporter plasmids pNF-κB-Luc, pIFN-β-Luc and pRL-TK were offered by Dr. Chunfu Zheng (School of Basic Medical Sciences, Fujian Medical University) (40–43). Ubiquitin expression plasmids pEFIRES-HA-Ub, pEFIRES-HA-Ub (K48) and pEFIRES-HA-Ub (K63) were provided by Dr. Jun Cui (School of Life Sciences, Sun Yat-sen University) (44). Other expression plasmids including IRAK1-HA (Dr. Hongyan Wang, Shanghai Institutes for Biological Sciences, Chinese Academy of Sciences) (45), pCMV-Flag-IRAK1 (Dr. Hongbin Shu, School of Life Sciences, Wuhan University), TRAF6-myc (Dr. Jiahuai Han, School of Life Sciences, Xiamen University) (46), Flag-tagged IRF3/5D (Dr. Rongtuan Lin, Department of Medicine, McGill University) (47), Flag-tagged IRF7/6D (Dr. John Hiscott, Lady Davis Institute, Jewish General Hospital) (48), pcDNA3.1-gD (Dr. Gary H. Cohen, University of Pennsylvania), pECFP-Golgi (Dr. Suzanne R. Pfeffer, Department of Biochemistry, Stanford University School of Medicine) (49), mCherry-KDEL (Dr. Lee H. Wong, Department of Biochemistry and Molecular Biology, Monash University; Dr. Philippe Collas, Institute of Basic Medical Sciences, University of Oslo) (50) and TOM70-CFP (Dr. Frits Kamp, Adolf-Butenandt-Institute, Ludwig-Maximilians-University) (51) were gifts from the providers shown as indicated.

### Plasmid Transfection, Indirect Immunofluorescence Assays (IFA), and Confocal Microscopy

COS-7 cells were grown overnight to 80% confluence on microscopy cover glass (NEST) placed in six well plate (Corning), then plasmid transfection and fluorescence microscopy experiments were carried out as described previously (40–43, 52). Briefly, COS-7 cells were transfected with the indicated plasmids DNA mixed with polyethylenimine (PEI) transfection reagent (Polysciences) according to the manufacturer's instructions. Twenty-four hours post-transfection, cells were fixed with 4% (v/v) paraformaldehyde (Beyotime Biotechnology) for 20 min at room temperature, washed for 3 times with PBS, and incubated with 0.2% Triton X-100 (Beyotime Biotechnology) for 30 min. Subsequently, cells were incubated with rabbit anti-gD pAb or mouse anti-Flag mAb, followed by incubation with tetramethyl rhodamine isocyanate (TRITC)-conjugated goat anti-rabbit IgG (Pierce) or fluorescein isothiocyanate (FITC)-conjugated goat anti-mouse IgG (Sigma-Aldrich), then stained with or without Nile Red (Sigma) for lipid droplets for 30 min, and finally stained with DAPI (4′6-diamidino-2-phe-nylindole) (Cell Signaling Technology) for 5 min when needed. Images were obtained with a confocal microscope (Axio-Imager-LSM-800, ZEISS, Germany) using a 600× oil-immersion objective. Each image represents a vast majority of the cells with similar subcellular distribution, and white color shows the co-localization of colors merged with green, blue and red, yellow color shows the co-localization of colors merged with green and red. All scale bars indicate 10 um.

### DLR Assays

The DLR assays were performed as described previously (38, 40–42, 53). In short, HEK293T cells were plated on 24 well dish (Corning) at a density of 1 × 10^5^ cells per well-overnight before transfection. Cells were then co-transfected with 100 ng of the indicated expression plasmid, 100 ng of IFN-β or NF-κB promoter reporter and 10 ng of pRL-TK (internal control) to normalize transfection efficiency. Twenty-four hours post-transfection, the luciferase activity was detected with a luciferase assay kit (Promega).

### Viral Proliferation

HSV-1 bacterial artificial chromosome (BAC) Luc (F strain, synchronally expressing firefly luciferase, and GFP fluorescent protein) was offered by Dr. Chunfu Zheng (54), which was reproduced and reposited in our lab. HEK293T cells were plated on 12 well-plate (Corning) overnight before infection, then HSV-1 BAC Luc was dissolved in DMEM medium and added to the cells at an MOI (multiplicity of infection) of 1. The virus was incubated for 1.5–2 h at 37°C in a 5% CO_2_ incubator and replaced with medium supplemented with 2% FBS to continue culture for the indicated times, then cells were harvested for luciferase reporter assays to determine the replication kinetics of HSV-1 (33, 34). Here, all experiments related to HSV-1 infection were carried out in the Biosafety Level II laboratory, and all operations were strictly performed in accordance with the biosafety operation requirements of Guangzhou Medical University.

### RNA Isolation and RT-qPCR

HEK293T cells cultured in 6 well plate were transfected with indicated amounts of expression plasmid. Twenty-four hours post-transfection, total RNA was extracted with TRIzol reagent (Invitrogen). Samples were then subjected to reverse transcription to cDNA with RT reagent (TSINGKE). The acquired cDNA was taken as a template for qPCR, to detect the expression of glyceraldehyde-3-phosphate dehydrogenase (GAPDH) (internal control) and IFN-β, using a qPCR instrument (BIO-RAD, CFX96). Primers used for GAPDH (forward primer 5′-AGG TCG GTG TGA ACG GAT TTG-3′ and reverse primer 5′-TGT AGA CCA TGT AGT TGA GGT CA-3′) and IFN-β (forward primer 5′-ATGACCAACAAGTGTCTCCTCC-3′ and reverse primer 5′- GGAATCCAAGCAAGTTGTAGCTC-3′) were referred to Bing Tian's report (55).

### Co-IP Assays

Co-IP assays were performed as previously described (40–42, 56–58). In brief, HEK293T cells were co-transfected with expression plasmids combination bearing EYFP, Flag, Myc or HA tag. Twenty-four hours post-transfection, transfected cells were infected with or without HSV-1 BAC Luc for 16 h, then cells were collected and lysed on ice with RIPA lysis buffer (Beyotime Biotechnology). For each immunoprecipitation (IP), an equivalent of lysate was incubated with mouse anti-Flag, anti-Myc or anti-HA mAb or non-specific control mouse antibody (IgG) and a 1:1 slurry of protein A/G PLUS-Agarose (Santa Cruz Biotechnology) at 4°C overnight. The Sepharose beads were then washed at least three times with lysis buffer added with 500 mM NaCl. Finally, immunoprecipitated proteins and cell lysates were subjected to immunoblotting (IB) assays with the indicated antibodies. The original IB results are shown in the Supplementary Material.

### Statistical Analysis

Statistical analyses were performed using Graphpad Prism 6 software. All data were normally distributed, and the homogeneity of variances was examined with Levene's test. As the samples were normally distributed and displayed homogenous variance, statistical analyses were performed using one-way ANOVA. In the event of a difference being present, Bonferroni-adjusted *post hoc* tests were performed to identify specific effect. Moreover, Student *t* test (unpaired two-tailed *t*-test) was used when needed. Data were expressed as means and standard deviations (mean ± SD) from three independent experiments, with significant differences marked on the figures. Significance levels were defined as ns, not significant, *P* > 0.05; ^*^P < 0.05; ^**^P < 0.01; ^***^P < 0.001; and ^****^P < 0.0001.

## Results

### gD Co-localizes With Viperin

**Figure 1:**
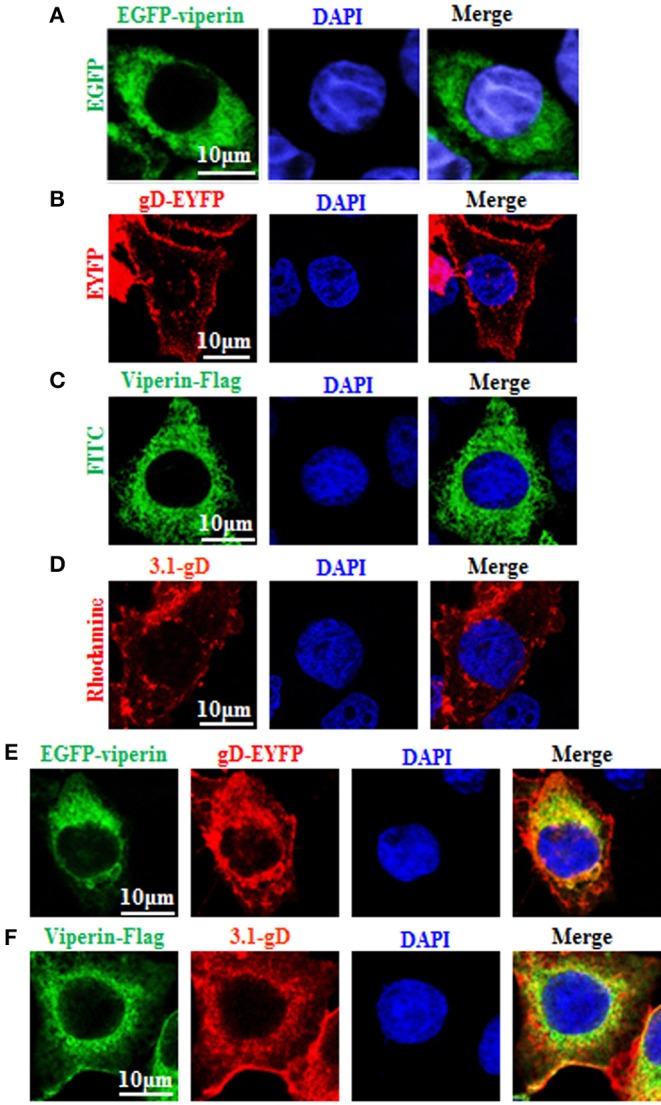
Co-localization of gD with viperin. **(A,B)** Subcellular localization of viperin and gD in live cells. COS-7 cells were transiently transfected with EGFP-viperin **(A)** or gD-EYFP **(B)** expression plasmid. Fluorescence image of EGFP-viperin fusion protein was presented in its original color green, and gD-EYFP fusion protein was presented in pseudo-color red. **(C,D)** Subcellular localization of viperin and gD in chemically fixation cells. Viperin-Flag **(C)** or 3.1-gD **(D)** expression plasmid was transfected into COS-7 cells, then IFA was performed with primary antibody mouse anti-Flag mAb or rabbit anti-gD pAb, and secondary antibody FITC-conjugated goat anti-mouse IgG or TRITC-conjugated goat anti-rabbit IgG, respectively. Fluorescence images of FITC-conjugated protein and TRITC-conjugated protein were presented in their original colors green and red, respectively. **(E)** Co-expression of EGFP-viperin and gD-EYFP in live cells. COS-7 cells were co-transfected with EGFP-viperin and gD-EYFP expression plasmids. Fluorescence images of fusion proteins were presented as indicated in **(A)**, and yellow color shows the co-localization of colors merged with green and red. **(F)** IFA analysis of COS-7 cells co-expressed with Viperin-Flag and 3.1-gD, with primary antibodies mouse anti-Flag mAb and rabbit anti-gD pAb, and secondary antibodies FITC-conjugated goat anti-mouse IgG and TRITC-conjugated goat anti-rabbit IgG. Twenty-four hours post-transfection, all the cells were stained with DAPI (blue) for 5 min, and analyzed with confocal microscopy. All of the photomicrographs were taken at a magnification of 600×. Each fluorescence image was representative of the vast majority of the cells observed. All scale bars indicate 10 um.

To find out which HSV-1 protein may interact with viperin, some HSV-1 encoded cytoplasmic localization proteins (2) were firstly screened, by co-transfection of viperin and HSV-1 protein expression plasmids and analyzing which HSV-1 protein can co-localize with viperin or alter its subcellular localization, and gD (US6), US4 (gG), and UL1 (gL) were identified. Our preliminary experiments found that there were significant differences in the interaction mechanisms among viperin-gD, viperin-US4 and viperin-UL1 (unpublished data). Therefore, the in-depth study of the interaction mechanisms between viperin and each protein of gD, US4, or UL1 would be an independent big project, and they need to be investigated separately. In addition, gD, US4 or UL1 encode glycoproteins, they (especially gD) play a very important role in the invasion of HSV-1. Accordingly, gD was firstly selected to investigate the potential interaction mechanism with viperin. To this end, pEGFP-viperin, pViperin-Flag, pgD-EYFP, or pcDNA3.1-gD expression plasmid was individually transfected into COS-7 cells to characterize their subcellular localizations in live cells or chemically fixed cells. As shown in Figure 1, viperin was absolutely distributed in the cytoplasm in cells transfected with EGFP-Viperin (Figure 1A) or Viperin-flag (Figure 1C) expression plasmid, and gD mainly exhibited nuclear membrane or cytoplasmic membrane localization in cells transfected with gD-EYFP (Figure 1B) or 3.1-gD (Figure 1D) expression plasmid, which are consistent with previous studies (59–61). In an attempt to pursue whether gD binds to viperin, EGFP-Viperin, and gD-EYFP expression plasmids were co-transfected into COS-7 cells to detect whether gD co-localizes with viperin, since co-localization experiment is one of the important and popular methods to detect the potential interaction between different proteins. As results, gD co-localized with viperin and predominantly accumulated at the perinuclear region (Figure 1E, yellow signal). Furthermore, IFA also proved the co-localization of gD and viperin at the perinuclear region (Figure 1F, yellow signal), confirming the potential interaction between gD and viperin.

### gD Interacts With Viperin

**Figure 2:**
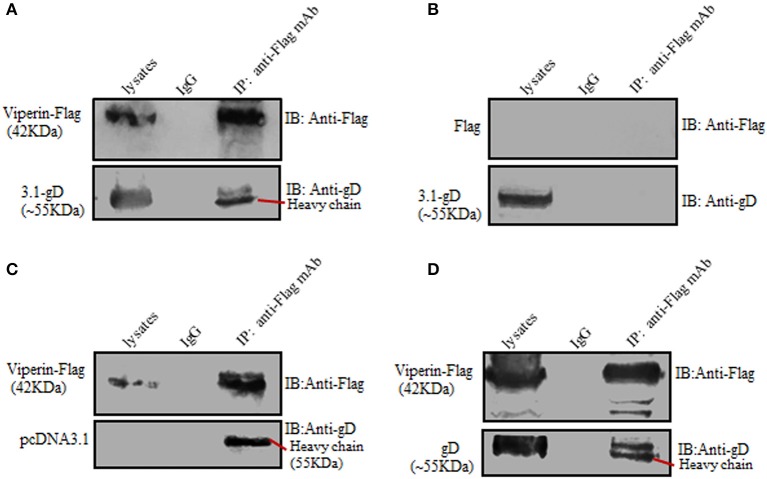
Verification of the interaction between gD and viperin. **(A–C)** Co-IP of viperin and gD from the lysates of transfected cells. HEK293T cells were co-transfected with plasmids combination pViperin-Flag/pcDNA3.1-gD **(A)**, Flag vector/pcDNA3.1-gD **(B)**, or pViperin-Flag/pcDNA3.1 **(C)**. Twenty-four hours post-transfection, cells were lysed and immunoprecipitated with mouse anti-Flag mAb or mouse IgG control. Immunoprecipitated proteins, as well as the cell lysates, were separated in denaturing 10% SDS-PAGE, and analyzed by IB with mouse anti-Flag mAb or mouse anti-gD mAb. **(D)** Co-IP of viperin and gD from the lysates of HSV-1 infected cells. HEK293T cells transfected with pViperin-Flag for 24 h were infected with HSV-1 BAC Luc at an MOI of 1 for 16 h. Then, cells were lysed and Co-IP assays were carried out, and analyzed by IB with mouse anti-Flag mAb or anti-gD mAb.

To further prove the interaction between gD and viperin, Co-IP assays were carried out. HEK293T cells were co-transfected with pcDNA3.1-gD and pViperin-Flag expression plasmids, then cell lysates were immunoprecipitated with anti-Flag mAb or non-specific control mouse IgG. As a result, gD was immunoprecipitated by Viperin-Flag with anti-Flag mAb (Figure 2A), whereas no such protein was immunoprecipitated with the control mouse IgG (Figure 2A). As negative controls, HEK293T cells were co-transfected with plasmids combination pcDNA3.1-gD/Flag vector (Figure 2B) or pViperin-Flag/pcDNA3.1 vector (Figure 2C). Then, cell lysates were immunoprecipitated with anti-Flag mAb or mouse IgG. Similarly, no target protein was immunoprecipitated by Flag vector (Figure 2B) or Viperin-Flag (Figure 2C), indicating gD could interact with viperin.

To continue determine the interaction between gD and viperin in the context of viral infection, HEK293T cells were transfected with pViperin-Flag expression plasmid, then infected with HSV-1 at an MOI of 1. Subsequently, cells were collected and Co-IP assays were performed. As shown in Figure 2D, gD again was immunoprecipitated by Viperin-Flag with anti-Flag mAb, whereas no such protein was immunoprecipitated with control mouse IgG, confirming gD could interact with viperin under physiological condition.

### Viperin Accumulates at Golgi Body and Lipid Droplets in the Presence of gD

**Figure 3:**
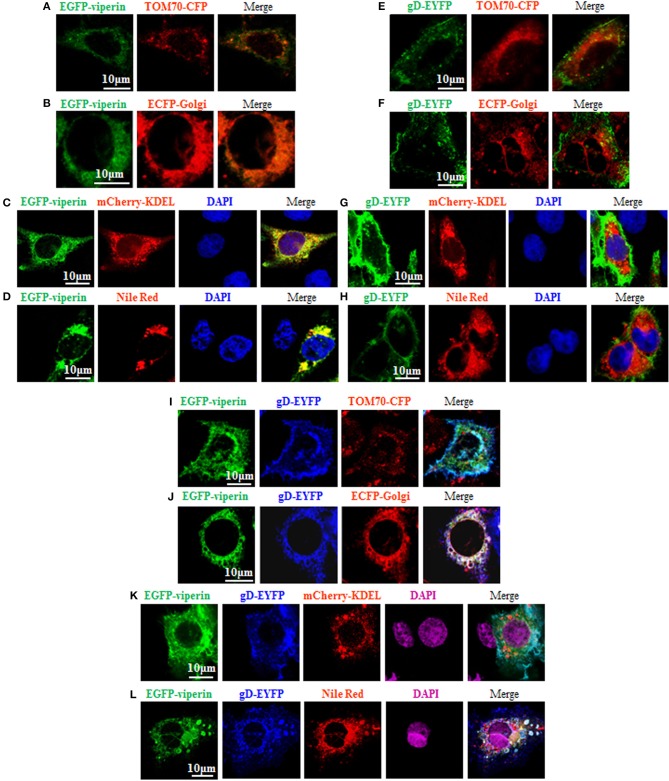
Viperin is accumulated at the Golgi body and lipid droplets in the presence of gD. **(A–H)** Expression plasmid of GFP-Viperin or gD-EYFP was transiently co-transfected with the subcellular marker expression plasmid of TOM70-CFP (Mitochondrial marker) **(A,E)**, ECFP-Glogi (Golgi marker) **(B,F)** or mCherry-KDEL (ER marker) **(C,G)** into COS-7 cells seeded on the coverslip in six well-plate. Twenty-four hours post-transfection, cells were stained with or without Nile Red (lipid droplets marker) **(D,H)** for 30 min and/or DAPI (blue) for 5 min when needed (only GFP-Viperin/mCherry-KDEL and GFP-Viperin/Nile Red panels can stained for DAPI, since the emission wavelength of CFP is similar with that of DAPI), then fixed and visualized with a confocal microscope using a 600× oil-immersion objective. Fluorescence image of fusion protein EGFP-viperin was presented in its original color green, subcellular organelle makers TOM70-CFP **(A,E)** and ECFP-Golgi **(B,F)** were presented in pseudo-color red, mCherry-KDEL **(C,G)** and Nile Red **(D,H)** were presented in their original color red, and gD-EYFP fusion protein was presented in pseudo-color green. Yellow color shows the co-localization of colors merged with green and red **(C,D)**. **(I–K)** Plasmids combination of gD-EYFP/EGFP-viperin were transiently co-transfected with the subcellular maker TOM70-CFP **(I)**, ECFP-Golgi **(J)**, or mCherry-KDEL **(K)** into COS-7 cells seeded on the coverslip in six well plate. Twenty-four hours post-transfection, cells were stained with or without Nile Red **(L)** for 30 min and/or DAPI (purple) for 5 min when needed. Then, confocal experiments were performed as described for **(A–H)**. Fluorescence image of fusion protein EGFP-viperin was presented in its original color green, subcellular makers TOM70-CFP **(I)** and ECFP-Golgi **(J)** were presented in pseudo-color red, mCherry-KDEL **(K)** and Nile Red **(L)** were presented in their original color red, and gD-EYFP fusion protein was presented in pseudo-color blue. White color shows the co-localization of colors merged with green, blue and red **(J,L)**. All scale bars indicate 10 um.

It is known that the N-terminal amphipathic a-helix is important for viperin to target to ER (60) and lipid droplets (62), and this subcellular localization is essential for suppressing viral replication (63). However, the vMIA-mediated mitochondria localization of viperin is favorable for HCMV replication (13). In order to probe the underlying mechanism of gD and viperin interaction, we continued to analyze whether gD can alter the normal localization of viperin. As control, GFP-Viperin or gD-EYFP was transiently co-transfected with the subcellular marker expression plasmid of TOM70-CFP (Mitochondrial marker) (Figures 3A,E), ECFP-Glogi (Golgi marker) (Figures 3B,F) or mCherry-KDEL (ER marker) (Figures 3C,G) into COS-7 cells, or cells were stained with Nile Red for lipid droplets (Figures 3D,H), and the cells were subsequently examined by confocal microscopy, to test the normal subcellular localizations of viperin and gD. As expected, viperin could co-localize with mCherry-KDEL (Figure 3C, yellow signal) and lipid droplets (Figure 3D, yellow signal), but not TOM70-CFP (Figure 3A) or ECFP-Glogi (Figure 3B) (13, 60, 62). However, gD-EYFP could not co-localize with all of the mentioned subcellular markers (Figures 3E–H). Then, expression plasmids combination of gD-EYFP/EGFP-viperin were transiently co-transfected with TOM70-CFP (Figure 3I), ECFP-Golgi (Figure 3J), or mCherry-KDEL (Figure 3K) into COS-7 cells, or cells were stained with Nile Red for lipid droplets. As results, co-expression of gD and viperin resulted in a pronounced co-localization with Golgi and lipid droplets markers (Figures 3J,L, white signal). Nevertheless, no obvious overlap area could be detected when gD and viperin were co-transfected with expression plasmid TOM70-CFP (Figure 3I) or mCherry-KDEL (Figure 3K). Therefore, viperin could accumulate at the Golgi apparatus and lipid droplets in the presence of gD.

### gD Facilitates IFN-β Activity in the Presence of Viperin

**Figure 4:**
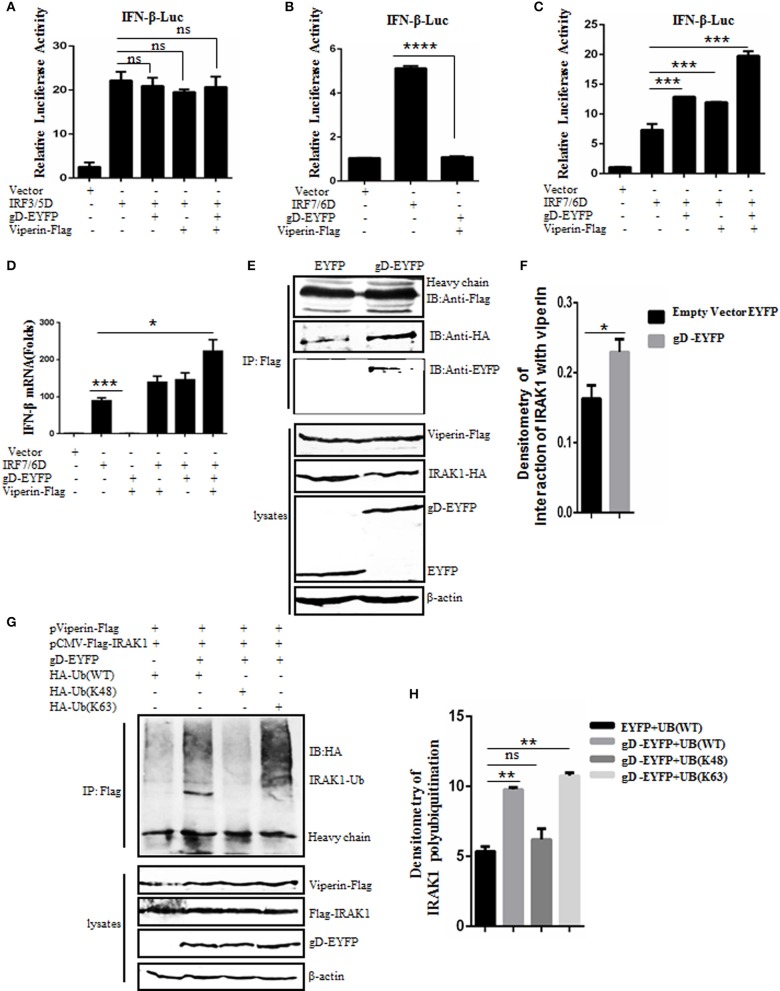
gD facilitates IRF7-mediated IFN-β promoter activity through enhancing the interaction of viperin with IRAK1 and increasing K63-linked polyubiquitination of IRAK1. **(A–C)** HEK293T cells were co-transfected with IFN-β-Luc reporter, pRL-TK and gD-EYFP or pViperin-Flag or plasmids combination of gD-EYFP and pViperin-Flag, with or without IRF3/5D **(A)** or IRF7/6D **(B,C)** expression plasmid. Twenty-four hours post-transfection, luciferase activity was analyzed. **(D)** HEK293T cells were co-transfected with the indicated plasmids as described in **(C)**, except for the reporter plasmids. Twenty-four hours post-transfection, RT-qPCR was performed to analyze the relative mRNA expression level of IFN-β. Data were expressed as means ± SD from three independent experiments. **(E,F)** HEK293T cells co-transfected with expression plasmids pViperin-Flag, IRAK1-HA and gD-EYFP, or EYFP control construct were harvested and immunoprecipitated with mouse anti-Flag mAb or non-specific mouse IgG, and IB analysis was probed with the indicated antibodies. Densitometry of the IRAK1 and viperin interaction bands were normalized to the loading control β-actin. **(G,H)** HA-tagged Ub (WT), Ub (K48), or Ub (K63) expression plasmid was co-transfected with plasmids combination of pCMV-Flag-IRAK1 and pViperin-Flag into HEK293T cells, with or without the presence of gD-EYFP. Twenty-four hours post-transfection, cells were collected, followed by Co-IP with mouse anti-Flag mAb and IB analysis with mouse anti-HA mAb. Densitometry of IRAK1 polyubiquitination bands were normalized to the loading control β-actin. Data were expressed as means ± SD from three independent experiments. Statistical analyses were performed using one-way ANOVA, except **(F)** using student *t* test. *P < 0.05; **P < 0.01; ***P < 0.001; and ****P < 0.0001.

It is reported that viperin can enhance TLR7/9-dependent production of IFN-I (29). To examine whether gD and viperin interaction is involved in the regulation of IFN-β expression, expression plasmid of gD-EYFP or pViperin-Flag or plasmids combination of gD-EYFP and pViperin-Flag were co-transfected with or without expression plasmid IRF3/5D or IRF7/6D into HEK293T cells, along with pIFN-β-Luc and pRL-TK reporter plasmids. As shown in Figure 4, both IRF3/5D and IRF7/6D alone could activate IFN-β expression, but no IFN-β activity was detected when HEK293T cells were only co-transfected with gD-EYFP and pViperin-Flag (Figures 4A–C). gD or viperin alone or combination of gD and viperin did not affect IRF3/5D-induced IFN-β activity (Figure 4A), however, gD or viperin alone could enhance IRF7/6D-induced IFN-β activity (Figure 4C). More importantly, the co-existence of gD and viperin activated a higher IFN-β promoter activity than that of gD or viperin (~2-fold) (Figure 4C). To further explore whether gD facilitates IFN-β activity through IRF7 in the presence of viperin, experiments were carried out as described in Figure 4C except for the reporter plasmids, and IFN-β mRNA accumulation was measured by RT-qPCR. As a result (Figure 4D), the change tendency of IFN-β mRNA was consistent with the DLR result shown in Figure 4C, suggesting the gD and viperin interaction could promote IRF7 mediated interferon expression.

### gD Enhances the Interaction Between Viperin and IRAK1

Saitoh's study shows that viperin can interact with the signal mediators IRAK1 and TRAF6, so as to recruit them to the lipid bodies, which can regulate TLR7- and TLR9-IRAK1 signaling axis to mediate the expression of functionally important immune molecules in plasmacytoid dendritic cell (29). To determine whether the gD and viperin interaction can affect the interaction between viperin and IRAK1, pViperin-Flag, and IRAK-HA expression plasmids were co-transfected with gD-EYFP or EYFP control vector into HEK293T cells, then cells were harvested and analyzed by Co-IP assays. In constrast to the EYFP control, the association of viperin and IRAK1 was enhanced in the presence of gD (Figures 4E,F).

### gD Increases the K63-Linked Polyubiquitination of IRAK1

It is shown that the viperin-related K63-linked polyubiquitination of IRAK1 is crucial for the TLR7- and TLR9-dependent IFN-β production (29). To probe whether gD and viperin interaction participates in the polyubiquitination of IRAK1, HA-tagged Ub (WT), Ub (K48), or Ub (K63) expression plasmid was co-transfected with plasmids pCMV-Flag-IRAK1 and pViperin-Flag into HEK293T cells, with or without the presence of gD-EYFP. Then, Co-IP assays were performed. As results, polyubiquitinated forms of IRAK1 were detected when cells were co-expressed with viperin and Ub (WT) (Figure 4G, lane 1, Figure 4H), and this polyubiquitination was reinforced in the presence of gD (Figure 4G, lane 2, Figure 4H). Notably, gD catalyzed IRAK1 polyubiquitination with the expression of Ub (K63) (Figure 4G, lane 4, Figure 4H), but not Ub (K48) (Figure 4G, lane 3, Figure 4H), indicating gD interacted with viperin to promote K63-linked IRAK1 polyubiquitination. In short, we demonstrated that gD could facilitate IFN-β production through enhancing the interaction of viperin with IRAK1 and increasing K63-linked polyubiquitination of IRAK1.

### gD Attenuates NF-κB Activity in the Presence of Viperin

**Figure 5:**
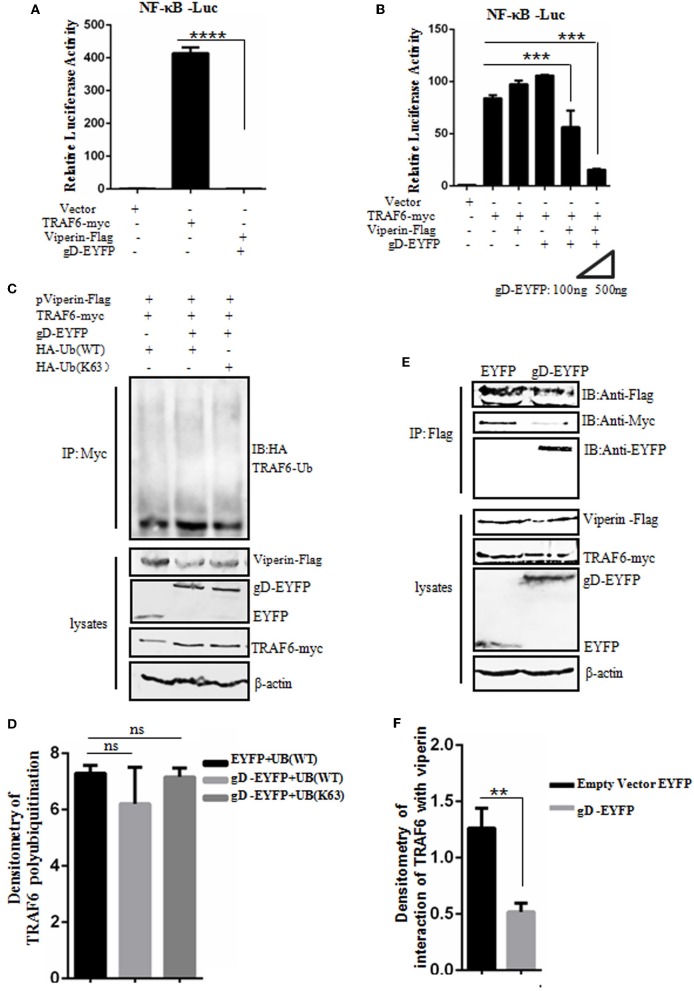
gD attenuates NF-κB activity by reducing the interaction between TRAF6 and viperin, but does not affect the polyubiquitination of TRAF6. **(A,B)** HEK293T cells were co-transfected with NF-κB-Luc reporter, pRL-TK and gD-EYFP or pViperin-Flag or plasmids combination of pViperin-Flag and gD-EYFP (with different amounts), with or without the presence of TRAF6-myc construct. Twenty-four hours post-transfection, luciferase activity was analyzed. Data were expressed as means ± SD from three independent experiments. **(C,D)** HEK293T cells were co-transfected with expression plasmids of TRAF6-myc, pViperin-Flag and HA-Ub (WT) or HA-Ub (K63), along with or without gD-EYFP expression plasmid. Twenty-four hours post-transfection, cells were collected, followed by Co-IP with mouse anti-Myc mAb and IB analysis with mouse anti-HA mAb. Densitometry of TRAF6 polyubiquitination bands were normalized to the loading control β-actin. **(E,F)** HEK293T cells co-transfected with expression plasmids of pViperin-Flag, TRAF6-myc and gD-EYFP or the control EYFP construct were harvested and Co-IPed with mouse anti-Flag mAb. IB analysis was probed with the indicated antibodies. Densitometry of the TRAF6 and viperin interaction bands were normalized to the loading control β-actin. Data were expressed as means ± SD from three independent experiments. Statistical analyses were performed using one-way ANOVA, except **(F)** using student *t* test. **P < 0.01; ***P < 0.001; and ****P < 0.0001.

Viperin is proved to be involved in the activation of NF-κB and AP-1 in T cells (64). To detect whether gD and viperin interaction also can modulate the NF-κB activity mediated by the key regulatory component of NF-κB signaling pathway, TRAF6 (11), expression plasmid gD-EYFP or pViperin-Flag or plasmids combination of gD-EYFP and pViperin-Flag were co-transfected with or without TRAF6-myc expression plasmid into HEK293T cells, along with pNF-κB-Luc and pRL-TK reporter plasmids. As results, overexpression of TRAF6 efficiently activated the NF-κB reporter, but no NF-κB activity was tested when HEK293T cells were only co-transfected with plasmids gD-EYFP and pViperin-Flag (Figure 5A). The expression of gD or viperin alone did not affect TRAF6-induced NF-κB reporter activity, however, gD and viperin combination significantly inhibited TRAF6-induced NF-κB reporter activity. Additionally, gD and viperin interaction constrained TRAF6-induced NF-κB promoter activity in a gD dose-dependent manner (Figure 5B), suggesting gD could modulate TRAF6-mediated NF-κB activity through viperin.

### gD Does Not Affect the Polyubiquitination of TRAF6

Polyubiquitination has emerged as an important regulatory mechanism in NF-κB signaling, and TRAF6 acts as a key substrate of K63-linked polyubiquitin chains in TNFR pathway, which serves as a mechanism to recruit TAK1 and IKK kinases and finally stimulate downstream NF-κB activation (65). To investigate whether gD and viperin interaction can affect the polyubiquitination of TRAF6, HEK293T cells were co-transfected with TRAF6-myc, pViperin-Flag and HA-Ub (WT) or HA-Ub (K63) constructs, along with or without gD-EYFP expression plasmid. Then, Co-IP assays were performed. As shown in Figure 5, no apparent difference of the TRAF6 polyubiquitination in the presence of gD or gD and viperin combination (Figures 5C,D), indicating gD and viperin interaction could not inhibit the polyubiquitination of TRAF6.

### gD Reduces the Interaction Between TRAF6 and Viperin

To further elucidate a clear molecular mechanism of how gD and viperin interaction inhibits NF-κB activity, pViperin-Flag, and TRAF6-myc expression plasmids were co-transfected into HEK293T cells, along with gD-EYFP or EYFP control plasmid. Then, Co-IP assays were carried out. In contrast to the EYFP control, gD significantly reduced the interaction between TRAF6 and viperin, suggesting gD could inhibit TRAF6-mediated NF-κB activity through competive binding viperin with TRAF6 (Figures 5E,F). Taken together, these results support that gD downregulated NF-κB activity by reducing the interaction between TRAF6 and viperin, but not affecting the polyubiquitination of TRAF6.

### gD Inhibits the Interaction Between IRAK1 and TRAF6 in the Presence of Viperin

**Figure 6:**
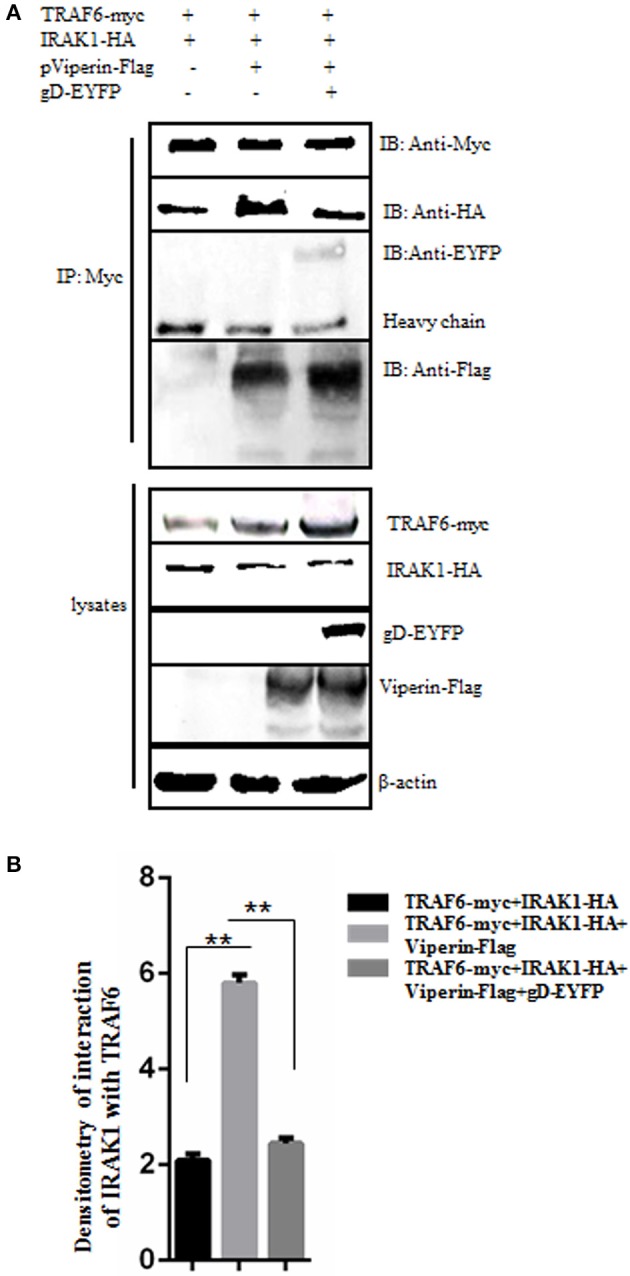
gD suppresses IRAK1 and TRAF6 interaction in the presence of viperin. **(A,B)** HEK293T cells were co-transfected with the expression plasmids of IRAK1-HA and TRAF6-myc, along with pViperin-Flag or plasmids combination of gD-EYFP and pViperin-Flag. Twenty-four hours post-transfection, cells were collected, and followed by Co-IP with mouse anti-Myc mAb. IB analysis was developed with the indicated antibodies. Densitometry of the IRAK1 and TRAF6 interaction bands were normalized to the loading control β-actin. Data were expressed as means ± SD from three independent experiments, and statistical analyses were performed using one-way ANOVA. **P < 0.01.

It is documented that the signal mediators IRAK1 and TRAF6 can interact with each other at the lipid bodies (29). To test whether gD and viperin interaction affects the interaction between IRAK1 and TRAF6, IRAK1-HA and TRAF6-myc expression plasmids were co-transfected into HEK293T cells, along with pViperin-Flag or expression plasmids combination of gD-EYFP and pViperin-Flag. Then, Co-IP assays were performed. As results, overexpression of viperin alone indeed induced significantly stronger interaction of IRAK1 and TRAF6 (Figure 6A, lane 2, Figure 6B), since viperin is critical for the recruitment of IRAK1 and TRAF6 to lipid bodies, which are the transfer points of TLR7 and TLR9 signaling pathways (29). However, the IRAK1 and TRAF6 interaction became weaker in the presence of gD and viperin (Figure 6A, lane 3, Figure 6B), which was similar to that of the negative control (Figure 6A, lane 1, Figure 6B), indicating gD and viperin interaction could impede the interaction between IRAK1 and TRAF6.

### gD and Viperin Interaction Inhibits HSV-1 Replication

**Figure 7:**
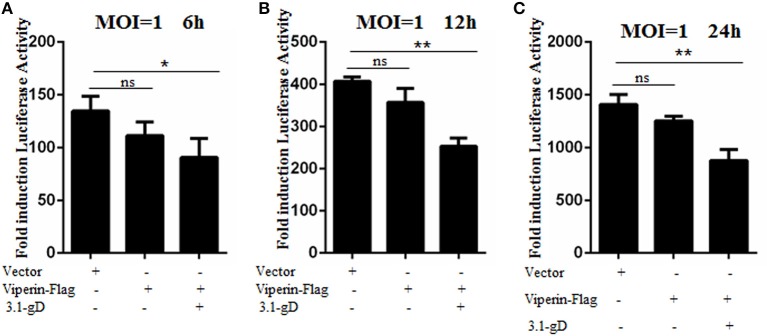
gD impairs HSV-1 replication in the presence of viperin. HEK293T cells were infected with HSV-1 BAC Luc at an MOI of 1 for 6 h **(A)**, 12 h **(B)**, or 24 h **(C)**, after transfection with plasmid pViperin-Flag or plasmids combination of 3.1-gD and pViperin-Flag for 12 h. Then, cells were harvested for luciferase reporter assays. Data were expressed as means ± SD from three independent experiments, and statistical analyses were carried out using one-way ANOVA. *P < 0.05; **P < 0.01.

In order to investigate the physiological significance of gD and viperin interaction during HSV-1 infection, HEK293T cells were mock-transfected or transfected with plasmid pViperin-Flag or plasmids combination of 3.1-gD and pViperin-Flag. Twelve hours post-transfection, cells were infected with HSV-1 BAC Luc at an MOI of 1 for 6, 12, or 24 h. Then, luciferase activity assays were performed to determine the replication kinetics of HSV-1. As shown in Figure 7, the luciferase activity gradually increased with the time extension of HSV-1 infection, and transfection with plasmid pViperin-Flag alone had no inhibitory effect on the HSV-1 propagation, which is consistent with our previous study (36). However, the HSV-1 proliferation was remarkably impaired when cells were co-transfected with 3.1-gD and pViperin-Flag expression plasmids. More importantly, this inhibitory trend was consistent at different time points of the infection (Figures 7A–C). Accordingly, these results indicated that the interaction between gD and viperin indeed could obstruct the reproduction of HSV-1.

## Discussion

Many viruses can induce the up-regulation of viperin during infection, and viperin is shown to have critical roles in inhibiting viral replication and facilitating TLR7- and TLR9-mediated production of IFN-I (29), yet its function can be dampened by some viruses (66). Our previous study has demonstrated that HSV-1 infection can not induce the up-regulation of viperin, since UL41 blocks the expression of viperin by reducing its mRNA accumulation (36). However, a small amount of viperin mRNA and its corresponding protein is not degraded (36), which is surprising and promotes us to probe whether other HSV-1 encoded proteins can interact with viperin to facilitate or inhibit the propagation of HSV-1. Accordingly, we utilized a simple and quick method at the beginning of screening, to analyze whether there are HSV-1 proteins (fused with EYFP) can co-localize with EGFP-viperin or change its normal subcellular localization. In the above cases, other methods were used to verify the potential interaction and interaction mechanism. In fact, we found that not only gD could co-localize with viperin, but also other HSV-1 proteins (unpublished data), indicating there are other potential interactions exist between HSV-1 proteins and viperin, which needed to be verified by further deep exploration. Certainly, there is bound to be a missed screening of interactions between HSV-1 proteins and viperin, although the method of fluorescence co-localization or localization change can help us to screen for potential interactions. Therefore, it is difficult for us to say when there is no interaction exist between HSV-1 proteins and viperin. Perhaps new interactions will be discovered by using other experimental methods.

In our previous experiment design, we had considered to detect the co-localization of endogenous gD and viperin during HSV-1 infection, but most of the endogenous viperin mRNA will be degraded by UL41 when HSV-1 infection. Western blot analysis showed that viperin was almost degraded, with very low remaining protein amount, hence in our previous experiment the expression of viperin could not be detected by IFA using endogenous viperin antibody (unpublished data), we therefore would not be able to study the interaction mechanism between gD and viperin without viperin overexpression (36). Actually, the interaction experiments were carried out under some physiological conditions, which were performed when HSV-1 infection using gD antibody (Figure 2). Furthermore, gD can not be deleted in the viral genome, since it is an essential protein for HSV-1 replication. Once deleted, HSV-1 can not proliferate. Thus, we can not study the interaction mechanism between gD and viperin during HSV-1 mutant (gD deletion or knockdown) infection (3, 8, 9), since it is difficult for us to determine whether the effect of gD-viperin interaction on the proliferation of HSV-1 (after gD knockdown or deletion) is caused by the decrease of gD and viperin interaction or the reduce of gD directly affects the propagation of HSV-1. Besides, various literatures have shown that the combination of plasmid transfection and viral infection (using virus protein specific antibody) is sufficient to validate the interaction between cellular protein and viral protein (35, 41, 67–69).

After screening, our confocal results found that gD could co-localize with viperin at the Golgi body and lipid droplets, and Co-IP results demonstrated that gD interacted with viperin. It is shown that MAVS can interact with viperin to act as a negative regulator of the interferon response (28). IRAK1 and TRAF6 are two other target proteins that can interplay with viperin and be recruited to the lipid bodies to induce the nuclear translocation of transcription factor IRF7 (29). Viperin also can interact with DENV-2 NS3 protein to restrict early DENV-2 RNA production/accumulation (30), and viperin can inhibit HCV replication via its interaction with NS5A (15). Surprisingly, viperin can enhance HCMV infection by interacting with the mitochondrial trifunctional protein, which mediates fatty acids β-oxidation to generate adenosine triphosphate (ATP) (13). Therefore, we wondered what is the effect of gD and viperin interaction.

It is well-known that toll-like receptors can recognize various pathogens (including viruses and bacteria) when they stimulate the innate immunity defense system (70), subsequently myeloid differentiation factor 88 (MyD88) is induced to recruit IRAK1 and form a complex through their respective death domains (71). Then, IRAK1 is phosphorylated and rapidly degraded in a proteasome-dependent manner, resulting in the down-regulation of IFN-I signaling and inflammatory responses (72). Upon receptor recognition, TLR2 dimerizes with either TLR1 or TLR6 and recruits MyD88. The next, TRAF6, which is an E3 ubiquitin ligase, catalyzes the synthesis of polyubiquitin chains bound to itself and TAK1, thereby activates TAK1 and leads to downstream NF-κB activation (73). Undoubtedly, IRAK1 and TRAF6 both are key regulatory components of the signaling pathway to mediate IFN-I production and canonical NF-κB-initiated cytokines. Accordingly, the expression of IFN and activation of NF-κB can be regulated through IRAK1 and TRAF6. For instance, newcastle disease virus facilitates K63-linked ubiquitination of IRAK1 to increase TLR7/9-dependent IFN-I production and subsequent expression of viperin (29). The nucleotide-binding domain and leucine-rich-repeat-containing (NLR) protein attenuates NF-κB activation through its interaction with the component of TRAF6 pathway (74). Therefore, we wanted to test whether gD and viperin interaction had any effect on the IFN-β or NF-κB pathway.

**Figure 8:**
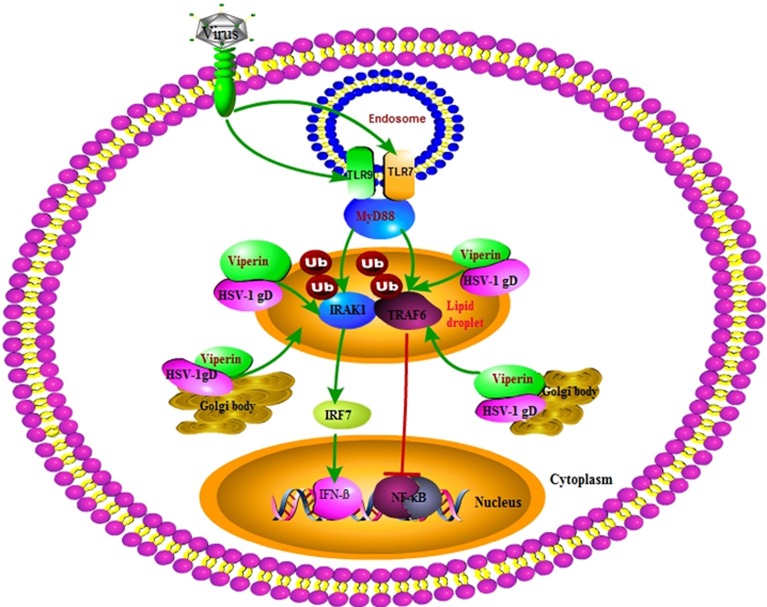
Overview of the molecular mechanism of HSV-1 gD and viperin interaction. HSV-1 gD can interact with viperin, and co-localize with it at the lipid droplets and Golgi body. The gD and viperin interaction facilitates IRF7-mediated IFN-β activity by promoting viperin and IRAK1 interaction and facilitating K63-linked IRAK1 ubiquitination, whereas gD attenuates TRAF6-induced NF-κB activity by inhibiting the viperin and TRAF6 interaction, but not affecting the polyubiquitination of TRAF6. Viperin alone promotes the interaction of IRAK1 and TRAF6, which is inhibited in the presence of gD and viperin. Eventually, gD and viperin interaction is corroborated to significantly inhibit the proliferation of HSV-1.

Our DLR assays showed that gD and viperin interaction could up-regulate IRF7 (but not IRF3) mediated IFN-β activity. Further Co-IP assays demonstrated that gD strengthened the interaction of viperin with IRAK1 and improved K63-linked IRAK1 polyubiquitination, suggesting the co-localization of gD with viperin at the Golgi body and lipid droplets can improve the antiviral ability of viperin. However, the presence of gD and viperin significantly inhibited TRAF6-mediated NF-κB activity in a gD dose-dependent manner. Co-IP results further showed gD reduced the interaction of TRAF6 with viperin, but not affected the ubiquitination of TRAF6 (Figure 8). Moreover, gD bound to viperin constrained the interaction between IRAK1 and TRAF6, which can interact with each other at the lipid bodies (29). Consequently, gD and viperin interaction was proved to restrain HSV-1 replication in physiological significance.

At the level of plasmid transfection, we had elucidated the specific interaction mechanism between gD and viperin, and we also analyzed and verified the effect of gD and viperin co-expression (overexpression) on HSV-1 proliferation. As mentioned above, the mRNA of viperin will be degraded by UL41 when HSV-1 infection, and the specific interaction mechanism between gD and viperin can not be studied after viperin is degraded. Therefore, we considered it was not necessary to carry out the viperin knockdown experiment, since viperin is actually degraded during HSV-1 infection.

Generally, a modest level of IFN-I expression is driven by the activation of NF-κB (75), however, excessive IFN-I expression can be restrained after NF-κB activation (76), this means that NF-κB can be activated but IFN-I is suppressed, and *vice versa*. Hence, gD interacts with viperin to inhibit NF-κB activity while stimulates IRF7-mediated IFN-I transcriptional activity is reasonable. This interaction is supposed to occur in HSV-1 lytic cycle rather than latent infection, since most of the HSV-1 proteins are silent when HSV-1 is latent in neurons, and only latency associated transcript (LAT) and a small amount of proteins are expressed (77).

When virus invades the cell, it activates a series of signaling pathways and then induces the expression of hundreds of ISGs to perform antiviral effects. Among them, viperin is an IFN-induced protein, which plays an important role in this process (11, 12). We supposes that in order to successfully infect the cells and establish effective replication, the HSV-1 encoded tegument protein UL41 can be released into the cytoplasm when HSV-1 invades cells, and the mRNAs of some ISGs (such as viperin) are degraded by UL41, so as to inhibit the host innate immunity and promote the proliferation of HSV-1 (36). However, HSV-1 has evolved very delicate mechanisms that if the functions of most of the ISGs are prohibited or HSV-1 continues to replicate strongly, it is bound to quickly cause the death of HSV-1 infected and adjacent cells, thereby HSV-1 does not have enough time to replicate, which is certainly not favorable for the survival of HSV-1. Accordingly, HSV-1 may take advantage of other encoded proteins (such as gD) to enhance the host's IFN response, by interacting with the pre-existing viperin or HSV-1-induced viperin, to compensate for the mRNA degradation of viperin, since the up-regulation of IFN will in turn promote the expression of viperin. Consequently, HSV-1 may balance the amount of viperin in a very sophisticated way, to regulate the relationship between host's innate immune status and its self-replication, but the specific mechanism needs to be further explored.

In conclusion, here we identified HSV-1 gD could interact with antiviral protein viperin, and co-localize with it at the Golgi body and lipid droplets. Our further results proved that gD and viperin interaction improved IRF7-mediated IFN-β activity to strengthen the antiviral ability of viperin, through enhancing the interaction between viperin and IRAK1, and increasing the K63-linked polyubiquitination of IRAK1. However, gD and viperin interaction could not affect the polyubiquitination of TRAF6, but decrease the interactions of TRAF6 with viperin and IRAK1, which finally inhibited the proliferation of HSV-1. It is noteworthy that the gD and viperin interaction may help us to explore and elucidate the roles of viperin and gD during HSV-1 infection.

## Data Availability Statement

The raw data supporting the conclusions of this manuscript will be made available by the authors, without undue reservation, to any qualified researcher.

## Author Contributions

MC and ML designed the research. ML, ZL, ZX, XZ, YW, HP, YL, XO, YD, YG, and WG performed the research. MC, ML, and ZL analyzed the data. DC and TP consulted and advised on the research. MC, ML, and ZL wrote and review the manuscript. All the authors read and approved the final manuscript.

### Conflict of Interest

TP was employed by company South China Vaccine Corporation Limited. The remaining authors declare that the research was conducted in the absence of any commercial or financial relationships that could be construed as a potential conflict of interest.

## Supplementary Material

Supplementary Material 1 Original immunoblotting results of Figure 2.

Supplementary Material 2 Original immunoblotting results of Figure 4E.

Supplementary Material 3 Original immunoblotting results of Figure 4G.

Supplementary Material 4 Original immunoblotting results of Figure 5C.

Supplementary Material 5 Original immunoblotting results of Figure 5E.

Supplementary Material 6 Original immunoblotting results of Figure 6A.

## References

[1] Loret S Guay G Lippé R. Comprehensive characterization of extracellular herpes simplex virus type 1 virions. J Virol. (2008) 82:8605–18. 10.1128/JVI.00904-08 18596102 PMC2519676

[2] Xing J Wang S Li Y Guo H Zhao L Pan W. Characterization of the subcellular localization of herpes simplex virus type 1 proteins in living cells. Med Microbiol Immunol. (2011) 200:61–8. 10.1007/s00430-010-0175-9 20949280

[3] Spear P Eisenberg R Cohen G. Three classes of cell surface receptors for alphaherpesvirus entry. Virology. (2000) 275:1–8. 10.1006/viro.2000.0529 11017782

[4] Cocchi F Menotti L Dubreuil P Lopez M Campadelli-Fiume G Cell-to-cell spread of wild-type herpes simplex virus type 1, but not of syncytial strains, is mediated by the immunoglobulin-like receptors that mediate virion entry, nectin1 (PRR1/HveC/HIgR) and nectin2 (PRR2/HveB). J Virol. (2000) 74:3909–17. 10.1128/JVI.74.8.3909-3917.2000 10729168 PMC111902

[5] Cocchi F Menotti L Mirandola P Lopez M Campadelli-Fiume G. The ectodomain of a novel member of the immunoglobulin subfamily related to the poliovirus receptor has the attributes of a bona fide receptor for herpes simplex virus types 1 and 2 in human cells. J Virol. (1998) 72:9992–10002. 9811737 10.1128/jvi.72.12.9992-10002.1998 PMC110516

[6] Shukla D Liu J Blaiklock P Shworak N Bai X Esko J. A novel role for 3-O-sulfated heparan sulfate in herpes simplex virus 1 entry. Cell. (1999) 99:13–22. 10.1016/S0092-8674(00)80058-6 10520990

[7] Carfí A Willis S Whitbeck J Krummenacher C Cohen G Eisenberg R. Herpes simplex virus glycoprotein D bound to the human receptor HveA. Mol Cell. (2001) 8:169–79. 10.1016/S1097-2765(01)00298-2 11511370

[8] Zhou G Galvan V Campadelli-Fiume G Roizman B. Glycoprotein D or J delivered in trans blocks apoptosis in SK-N-SH cells induced by a herpes simplex virus 1 mutant lacking intact genes expressing both glycoproteins. J Virol. (2000) 74:11782–91. 10.1128/JVI.74.24.11782-11791.2000 11090178 PMC112461

[9] Mehmood A Kaushik AC Wei DQ. Prediction and validation of potent peptides against herpes simplex virus type 1 via immunoinformatic and systems biology approach. Chem Biol Drug Des. (2019) 94:1868–83. 10.1111/cbdd.13602 31437863

[10] Carmichael JC Starkey J Zhang D Sarfo A Chadha P Wills JW. Glycoprotein D of HSV-1 is dependent on tegument protein UL16 for packaging and contains a motif that is differentially required for syncytia formation. Virology. (2019) 527: 64–76. 10.1016/j.virol.2018.09.018 30465930 PMC6400489

[11] Su C Zhan G Zheng C. Evasion of host antiviral innate immunity by HSV-1, an update. Virol J. (2016) 13:38. 10.1186/s12985-016-0495-5 26952111 PMC4782282

[12] Siegal FP Kadowaki N Shodell M Fitzgerald-Bocarsly PA Shah K Ho S. The nature of the principal type 1 interferon-producing cells in human blood. Science. (1999) 284:1835–7. 10.1126/science.284.5421.1835 10364556

[13] Seo J Yaneva R Hinson E Cresswell P. Human cytomegalovirus directly induces the antiviral protein viperin to enhance infectivity. Science. (2011) 332:1093–7. 10.1126/science.1202007 21527675

[14] Helbig K Lau D Semendric L Harley H Beard M. Analysis of ISG expression in chronic hepatitis C identifies viperin as a potential antiviral effector. Hepatology. (2005) 42:702–10. 10.1002/hep.20844 16108059

[15] Helbig K Eyre N Yip E Narayana S Li K Fiches G. The antiviral protein viperin inhibits hepatitis C virus replication via interaction with nonstructural protein 5A. Hepatology. (2011) 54:1506–17. 10.1002/hep.24542 22045669 PMC3207276

[16] Chin K Cresswell P. Viperin (cig5), an IFN-inducible antiviral protein directly induced by human cytomegalovirus. Proc Natl Acad Sci USA. (2001) 98:15125–30. 10.1073/pnas.011593298 11752458 PMC64994

[17] Boudinot P Riffault S Salhi S Carrat C Sedlik C Mahmoudi N. Vesicular stomatitis virus and pseudorabies virus induce a vig1/cig5 homologue in mouse dendritic cells via different pathways. J Gen Virol. (2000) 81(Pt 11):2675–82. 10.1099/0022-1317-81-11-2675 11038379

[18] Chan Y Chang T Liao C Lin Y. The cellular antiviral protein viperin is attenuated by proteasome-mediated protein degradation in Japanese encephalitis virus-infected cells. J Virol. (2008) 82:10455–64. 10.1128/JVI.00438-08 18768981 PMC2573197

[19] Szretter K Brien J Thackray L Virgin H Cresswell P Diamond M. The interferon-inducible gene viperin restricts West Nile virus pathogenesis. J Virol. (2011) 85:11557–66. 10.1128/JVI.05519-11 21880757 PMC3209274

[20] White L Sali T Alvarado D Gatti E Pierre P Streblow D. Chikungunya virus induces IPS-1-dependent innate immune activation and protein kinase R-independent translational shutoff. J Virol. (2011) 85:606–20. 10.1128/JVI.00767-10 20962078 PMC3014158

[21] Proud D Turner R Winther B Wiehler S Tiesman J Reichling T. Gene expression profiles during *in vivo* human rhinovirus infection: insights into the host response. Am J Respir Crit Care Med. (2008) 178:962–8. 10.1164/rccm.200805-670OC 18658112

[22] Khaiboullina S Rizvanov A Holbrook M St. Jeor S. Yellow fever virus strains Asibi and 17D-204 infect human umbilical cord endothelial cells and induce novel changes in gene expression. Virology. (2005) 342:167–76. 10.1016/j.virol.2005.07.035 16150475

[23] Hinson ER Joshi NS Chen JH Rahner C Jung YW Wang X. Viperin is highly induced in neutrophils and macrophages during acute and chronic lymphocytic choriomeningitis virus infection. J Immunol. (2010) 184:5723–31. 10.4049/jimmunol.0903752 20410488 PMC3883313

[24] Fink J Gu F Ling L Tolfvenstam T Olfat F Chin K. Host gene expression profiling of dengue virus infection in cell lines and patients. PLoS Negl Trop Dis. (2007) 1:e86. 10.1371/journal.pntd.0000086 18060089 PMC2100376

[25] Tang H Lu Z Wei X Zhong T Zhong Y Ouyang L. Viperin inhibits rabies virus replication via reduced cholesterol and sphingomyelin and is regulated upstream by TLR4. Sci Rep. (2016) 6:30529. 10.1038/srep30529 27456665 PMC4960569

[26] Bajimaya S Frankl T Hayashi T Takimoto T. Cholesterol is required for stability and infectivity of influenza A and respiratory syncytial viruses. Virology. (2017) 510:234–41. 10.1016/j.virol.2017.07.024 28750327 PMC5571833

[27] Wang X Hinson ER Cresswell P. The interferon-inducible protein viperin inhibits influenza virus release by perturbing lipid rafts. Cell Host Microbe. (2007) 2:96–105. 10.1016/j.chom.2007.06.009 18005724

[28] Hee J Cresswell P. Viperin interaction with mitochondrial antiviral signaling protein (MAVS) limits viperin-mediated inhibition of the interferon response in macrophages. PLoS ONE. (2017) 12:e0172236. 10.1371/journal.pone.0172236 28207838 PMC5313200

[29] Saitoh T Satoh T Yamamoto N Uematsu S Takeuchi O Kawai T. Antiviral protein Viperin promotes Toll-like receptor 7- and Toll-like receptor 9-mediated type I interferon production in plasmacytoid dendritic cells. Immunity. (2011) 34:352–63. 10.1016/j.immuni.2011.03.010 21435586

[30] Helbig K Carr J Calvert J Wati S Clarke J Eyre N. Viperin is induced following dengue virus type-2 (DENV-2) infection and has anti-viral actions requiring the C-terminal end of viperin. PLoS Negl Trop Dis. (2013) 7:e2178. 10.1371/journal.pntd.0002178 23638199 PMC3630087

[31] Kadowaki N Antonenko S Lau JY Liu YJ. Natural interferon alpha/beta-producing cells link innate and adaptive immunity. J Exp Med. (2000) 192:219–26. 10.1084/jem.192.2.219 10899908 PMC2193254

[32] Zheng C. Evasion of Cytosolic DNA-stimulated innate immune responses by herpes simplex virus 1. J Virol. (2018) 92:JVI.00099–17. 10.1128/JVI.00099-17 29298887 PMC5827395

[33] Wang S Wang K Lin R Zheng C. Herpes simplex virus 1 serine/threonine kinase US3 hyperphosphorylates IRF3 and inhibits beta interferon production. J Virol. (2013) 87:12814–27. 10.1128/JVI.02355-13 24049179 PMC3838156

[34] Wang S Wang K Li J Zheng C. Herpes simplex virus 1 ubiquitin-specific protease UL36 inhibits beta interferon production by deubiquitinating TRAF3. J Virol. (2013) 87:11851–60. 10.1128/JVI.01211-13 23986588 PMC3807349

[35] Xing J Ni L Wang S Wang K Lin R Zheng C. Herpes simplex virus 1-encoded tegument protein VP16 abrogates the production of beta interferon (IFN) by inhibiting NF-κB activation and blocking IFN regulatory factor 3 to recruit its coactivator CBP. J Virol. (2013) 87:9788–801. 10.1128/JVI.01440-13 23824799 PMC3754106

[36] Shen G Wang K Wang S Cai M Li M Zheng C. Herpes simplex virus 1 counteracts viperin via its virion host shutoff protein UL41. J Virol. (2014) 88:12163–6. 10.1128/JVI.01380-14 25078699 PMC4178720

[37] Tanaka M Kagawa H Yamanashi Y Sata T Kawaguchi Y. Construction of an excisable bacterial artificial chromosome containing a full-length infectious clone of herpes simplex virus type 1: viruses reconstituted from the clone exhibit wild-type properties *in vitro* and *in vivo*. J Virol. (2003) 77:1382–91. 10.1128/JVI.77.2.1382-1391.2003 12502854 PMC140785

[38] Cai M Li M Wang K Wang S Lu Q Yan J. The herpes simplex virus 1-encoded envelope glycoprotein B activates NF-κB through the Toll-like receptor 2 and MyD88/TRAF6-dependent signaling pathway. PLoS ONE. (2013) 8:e54586. 10.1371/journal.pone.0054586 23382920 PMC3557241

[39] Cai M Chen D Zeng Z Yang H Jiang S Li X. Characterization of the nuclear import signal of herpes simplex virus 1 UL31. Arch Virol. (2016) 161:2379–85. 10.1007/s00705-016-2910-z 27276975

[40] Zhang D Su C Zheng C. Herpes simplex virus 1 serine protease VP24 blocks the DNA-sensing signal pathway by abrogating activation of interferon regulatory factor 3. J Virol. (2016) 90:5824–29. 10.1128/JVI.00186-16 27076640 PMC4886774

[41] Xu H Su C Pearson A Mody CH Herpes simplex virus 1 UL24 abrogates the DNA sensing signal pathway by inhibiting NF-kB activation. J Virol. (2017) 91:e00025–17. 10.1128/JVI.00025-17 28100608 PMC5355614

[42] Ye R Su C Xu H Zheng C Herpes simplex virus 1 ubiquitin-specific protease UL36 abrogates NF-kB activation in DNA sensing signal pathway. J Virol. (2017) 91:e02417–16. 10.1128/JVI.02417-16 28031360 PMC5309955

[43] Su C Zheng C. Herpes simplex virus 1 abrogates the cGAS/STING-mediated cytosolic DNA-sensing pathway via its virion host shutoff protein, UL41. J Virol. (2017) 91:e02414–16. 10.1128/JVI.02414-16 28077645 PMC5331819

[44] Lin M Zhao Z Yang Z Meng Q Tan P Xie W. USP38 inhibits type I interferon signaling by editing TBK1 ubiquitination through NLRP4 signalosome. Mol Cell. (2016) 64:267–81. 10.1016/j.molcel.2016.08.029 27692986

[45] Li W Xiao J Zhou X Xu M Hu C Xu X. STK4 regulates TLR pathways and protects against chronic inflammation-related hepatocellular carcinoma. J Clin Invest. (2015) 125:4239–54. 10.1172/JCI81203 26457732 PMC4639976

[46] Ge B Gram H Di Padova F Huang B New L Ulevitch R. MAPKK-independent activation of p38alpha mediated by TAB1-dependent autophosphorylation of p38alpha. Science. (2002) 295:1291–4. 10.1126/science.1067289 11847341

[47] Lin R Genin P Mamane Y Sgarbanti M Battistini A Harrington W. HHV-8 encoded vIRF-1 represses the interferon antiviral response by blocking IRF-3 recruitment of the CBP/p300 coactivators. Oncogene. (2001) 20:800–11. 10.1038/sj.onc.1204163 11314014

[48] Paz S Vilasco M Arguello M Sun Q Lacoste J Nguyen T. Ubiquitin-regulated recruitment of IkappaB kinase epsilon to the MAVS interferon signaling adapter. Mol Cell Biol. (2009) 29:3401–12. 10.1128/MCB.00880-08 19380491 PMC2698723

[49] Barbero P Bittova L Pfeffer S. Visualization of Rab9-mediated vesicle transport from endosomes to the trans-Golgi in living cells. J Cell Biol. (2002) 156:511–8. 10.1083/jcb.200109030 11827983 PMC2173336

[50] Avalos A Kirak O Oelkers J Pils M Kim Y Ottinger M. Cell-specific TLR9 trafficking in primary APCs of transgenic TLR9-GFP mice. J Immunol. (2013) 190:695–702. 10.4049/jimmunol.1202342 23241879 PMC3539690

[51] Kamp F Exner N Lutz A Wender N Hegermann J Brunner B. Inhibition of mitochondrial fusion by α-synuclein is rescued by PINK1, Parkin and DJ-1. EMBO J. (2010) 29:3571–89. 10.1038/emboj.2010.223 20842103 PMC2964170

[52] Cai M Liao Z Chen T Wang P Zou X Wang Y. Characterization of the subcellular localization of Epstein-Barr virus encoded proteins in live cells. Oncotarget. (2017) 8:70006–34. 10.18632/oncotarget.19549 29050259 PMC5642534

[53] Huang J You H Su C Li Y Chen S Zheng C. Herpes simplex virus 1 tegument protein VP22 abrogates cGAS/STING-mediated antiviral innate immunity. J Virol. (2018) 92:e00841–18. 10.1128/JVI.00841-18 29793952 PMC6052299

[54] Li Y Wang S Zhu H Zheng C. Cloning of the herpes simplex virus type 1 genome as a novel luciferase-tagged infectious bacterial artificial chromosome. Arch Virol. (2011) 156:2267–72. 10.1007/s00705-011-1094-9 21894520

[55] Tian B Zhao Y Kalita M Edeh CB Paessler S Casola A. CDK9-dependent transcriptional elongation in the innate interferon-stimulated gene response to respiratory syncytial virus infection in airway epithelial cells. J Virol. (2013) 87:7075–92. 10.1128/JVI.03399-12 23596302 PMC3676079

[56] Xing J Wang S Lin F Pan W Hu C Zheng C. Comprehensive characterization of interaction complexes of herpes simplex virus type 1 ICP22, UL3, UL4, and UL20.5. J Virol. (2011) 85:1881–6. 10.1128/JVI.01730-10 21147926 PMC3028915

[57] Cai M Jiang S Zeng Z Li X Mo C Yang Y. Probing the nuclear import signal and nuclear transport molecular determinants of PRV ICP22. Cell Biosci. (2016) 6:3. 10.1186/s13578-016-0069-7 26816613 PMC4727382

[58] Cai M Si J Li X Zeng Z Li M. Characterization of the nuclear import mechanisms of HSV-1 UL31. Biol Chem. (2016) 397:555–61. 10.1515/hsz-2015-0299 26854290

[59] Whiteley A Bruun B Minson T Browne H. Effects of targeting herpes simplex virus type 1 gD to the endoplasmic reticulum and trans-Golgi network. J Virol. (1999) 73:9515–20. 10516060 10.1128/jvi.73.11.9515-9520.1999 PMC112986

[60] Hinson E Cresswell P. The N-terminal amphipathic alpha-helix of viperin mediates localization to the cytosolic face of the endoplasmic reticulum and inhibits protein secretion. J Biol Chem. (2009) 284:4705–12. 10.1074/jbc.M807261200 19074433 PMC2640954

[61] Fujimoto T Parton R. Not just fat: the structure and function of the lipid droplet. Cold Spring Harb Perspect Biol. (2011) 3:a004838. 10.1101/cshperspect.a004838 21421923 PMC3039932

[62] Hinson E Cresswell P. The antiviral protein, viperin, localizes to lipid droplets via its N-terminal amphipathic alpha-helix. Proc Natl Acad Sci USA. (2009) 106:20452–7. 10.1073/pnas.0911679106 19920176 PMC2778571

[63] Upadhyay A Vonderstein K Pichlmair A Stehling O Bennett K Dobler G. Viperin is an iron-sulfur protein that inhibits genome synthesis of tick-borne encephalitis virus via radical SAM domain activity. Cell Microbiol. (2014) 16:834–48. 10.1111/cmi.12241 24245804

[64] Qiu L Cresswell P Chin K. Viperin is required for optimal Th2 responses and T-cell receptor-mediated activation of NF-kappaB and AP-1. Blood. (2009) 113:3520–9. 10.1182/blood-2008-07-171942 19047684

[65] Shembade N Harhaj E. Elucidating dynamic protein-protein interactions and ubiquitination in NF-κB signaling pathways. Methods Mol Biol. (2015) 1280:283–95. 10.1007/978-1-4939-2422-6_16 25736755

[66] Lee H Lee J Park Y. E7 protein of cutaneous human papillomavirus attenuates viperin expression in human keratinocytes. J Dermatol Sci. (2017) 87:91–94. 10.1016/j.jdermsci.2017.02.001 28242342

[67] Zhang J Wang S Wang K Zheng C Herpes simplex virus 1 DNA polymerase processivity factor UL42 inhibits TNF-alpha-induced NF-kB activation by interacting with p65/RelA and p50/NF-kB1. Med Microbiol Immunol. (2013) 202:313–25. 10.1007/s00430-013-0295-0 23636254

[68] Zhang J Wang K Wang S Zheng C Herpes simplex virus 1 E3 ubiquitin ligase ICP0 protein inhibits tumor necrosis factor alpha-induced NF-kB activation by interacting with p65/RelA and p50/NF-kB1. J Virol. (2013) 87:12935–48. 10.1128/JVI.01952-13 24067962 PMC3838126

[69] Xing J Wang S Lin R Mossman KL Zheng C. Herpes simplex virus 1 tegument protein US11 downmodulates the RLR signaling pathway via direct interaction with RIG-I and MDA-5. J Virol. (2012) 86:3528–40. 10.1128/JVI.06713-11 22301138 PMC3302539

[70] Akira S Uematsu S Takeuchi O. Pathogen recognition and innate immunity. Cell. (2006) 124:783–801. 10.1016/j.cell.2006.02.015 16497588

[71] Loiarro M Gallo G Fantò N De Santis R Carminati P Ruggiero V. Identification of critical residues of the MyD88 death domain involved in the recruitment of downstream kinases. J Biol Chem. (2009) 284:28093–103. 10.1074/jbc.M109.004465 19679662 PMC2788860

[72] Yamin TT Miller DK. The interleukin-1 receptor-associated kinase is degraded by proteasomes following its phosphorylation. J Biol Chem. (1997) 272:21540–7. 10.1074/jbc.272.34.21540 9261174

[73] Oliveira-Nascimento L Massari P Wetzler LM. The role of TLR2 in infection and immunity. Front Immunol. (2012) 3:79. 10.3389/fimmu.2012.00079 22566960 PMC3342043

[74] Allen I Moore C Schneider M Lei Y Davis B Scull M. NLRX1 protein attenuates inflammatory responses to infection by interfering with the RIG-I-MAVS and TRAF6-NF-κB signaling pathways. Immunity. (2011) 34:854–65. 10.1016/j.immuni.2011.03.026 21703540 PMC3166771

[75] Cotter C Kim W Nguyen M Yount J López C Blaho J. The virion host shutoff protein of herpes simplex virus 1 blocks the replication-independent activation of NF-κB in dendritic cells in the absence of type I interferon signaling. J Virol. (2011) 85:12662–72. 10.1128/JVI.05557-11 21937652 PMC3209407

[76] Wang L Wang Y Zhao J Ren J Hall K Moorman J. The linear ubiquitin assembly complex modulates latent membrane protein 1 activation of NF-κB and interferon regulatory factor 7. J Virol. (2017) 91:e01138–16. 10.1128/JVI.01138-16 27903798 PMC5286870

[77] Evans CM Kudesia G McKendrick M. Management of herpesvirus infections. Int J Antimicrob Agents. (2013) 42:119–28. 10.1016/j.ijantimicag.2013.04.023 23820015

