# Supplementary figures and images for: The Interaction Mechanism Between Herpes Simplex Virus 1 Glycoprotein D and Host Antiviral Protein Viperin
Source: Front Immunol. 2019 Dec 11;10:2810. doi: 10.3389/fimmu.2019.02810 (PMC6917645; doi:10.3389/fimmu.2019.02810)

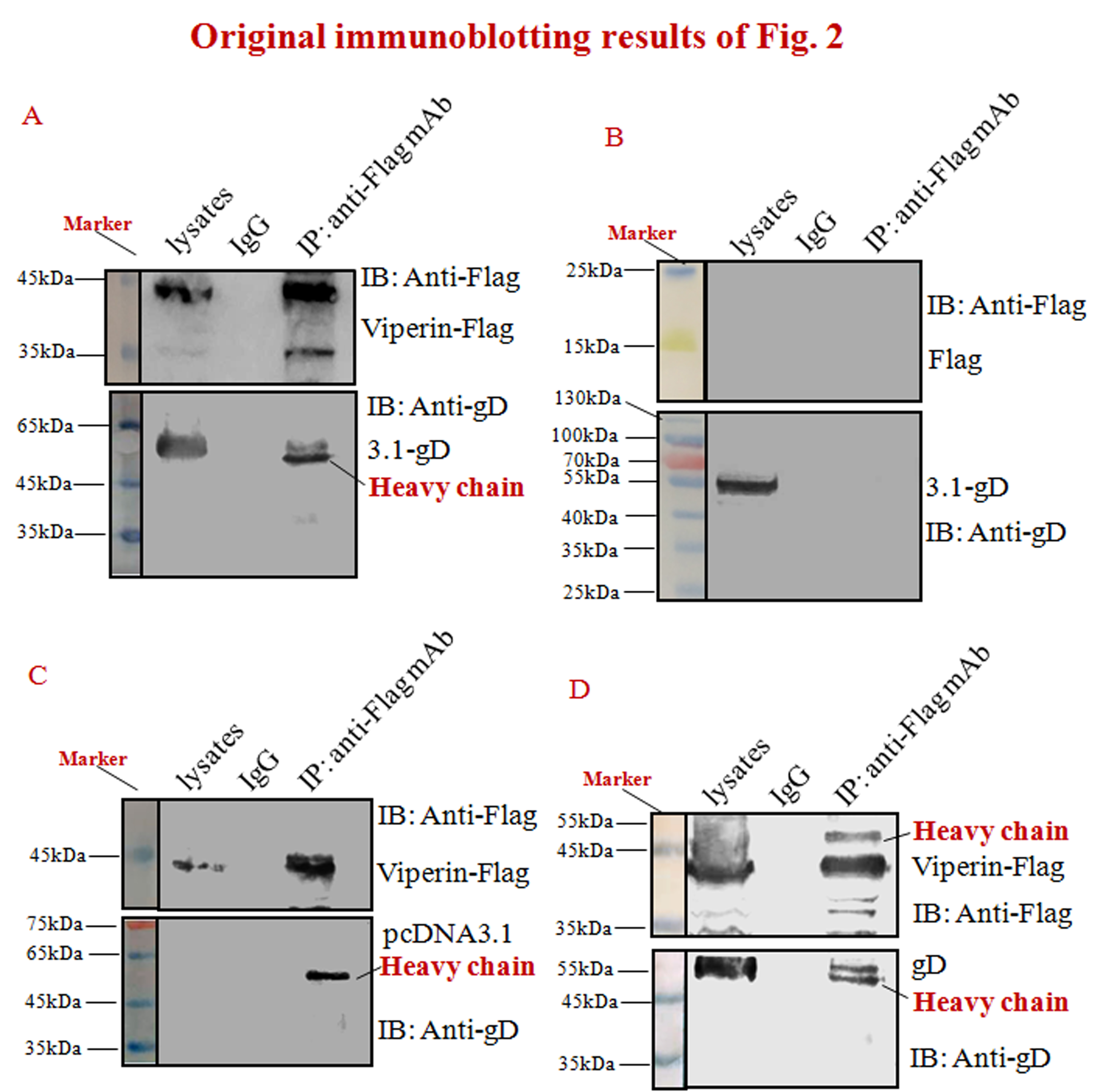

Supplement: Supplementary Material 1 — Original immunoblotting results of Figure 2. [file Image_1.jpg]

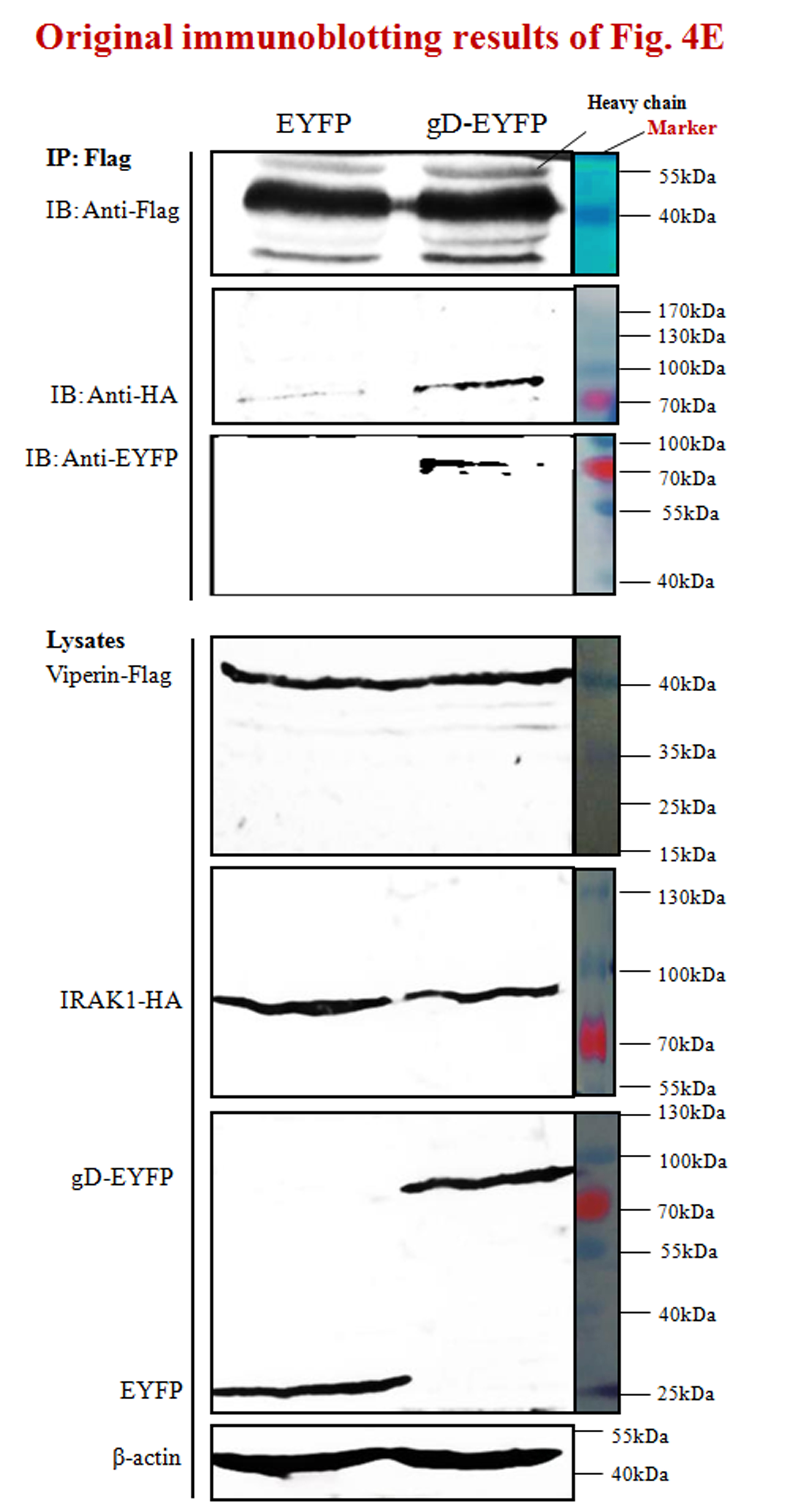

Supplement: Supplementary Material 2 — Original immunoblotting results of Figure 4E. [file Image_2.jpg]

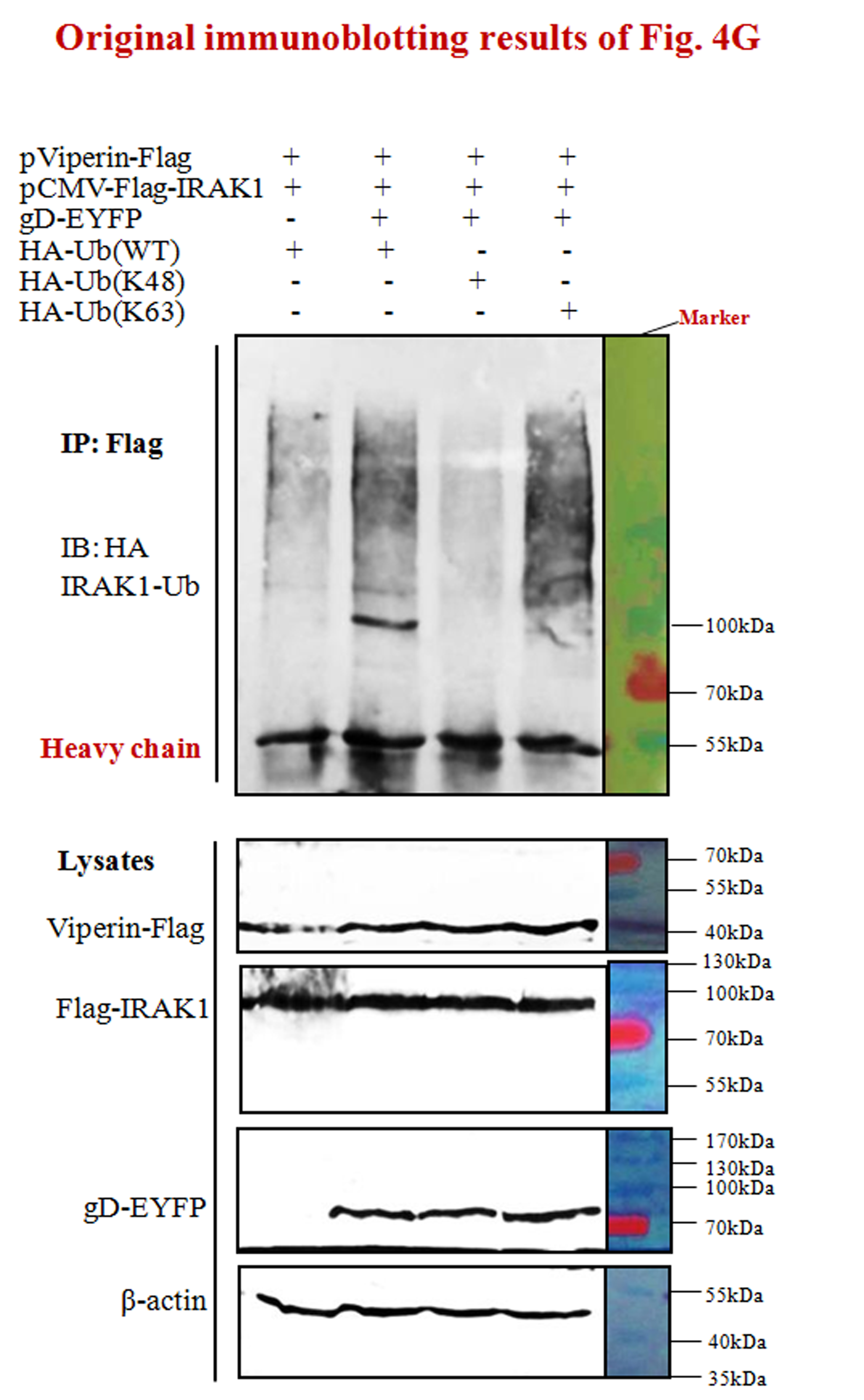

Supplement: Supplementary Material 3 — Original immunoblotting results of Figure 4G. [file Image_3.jpg]

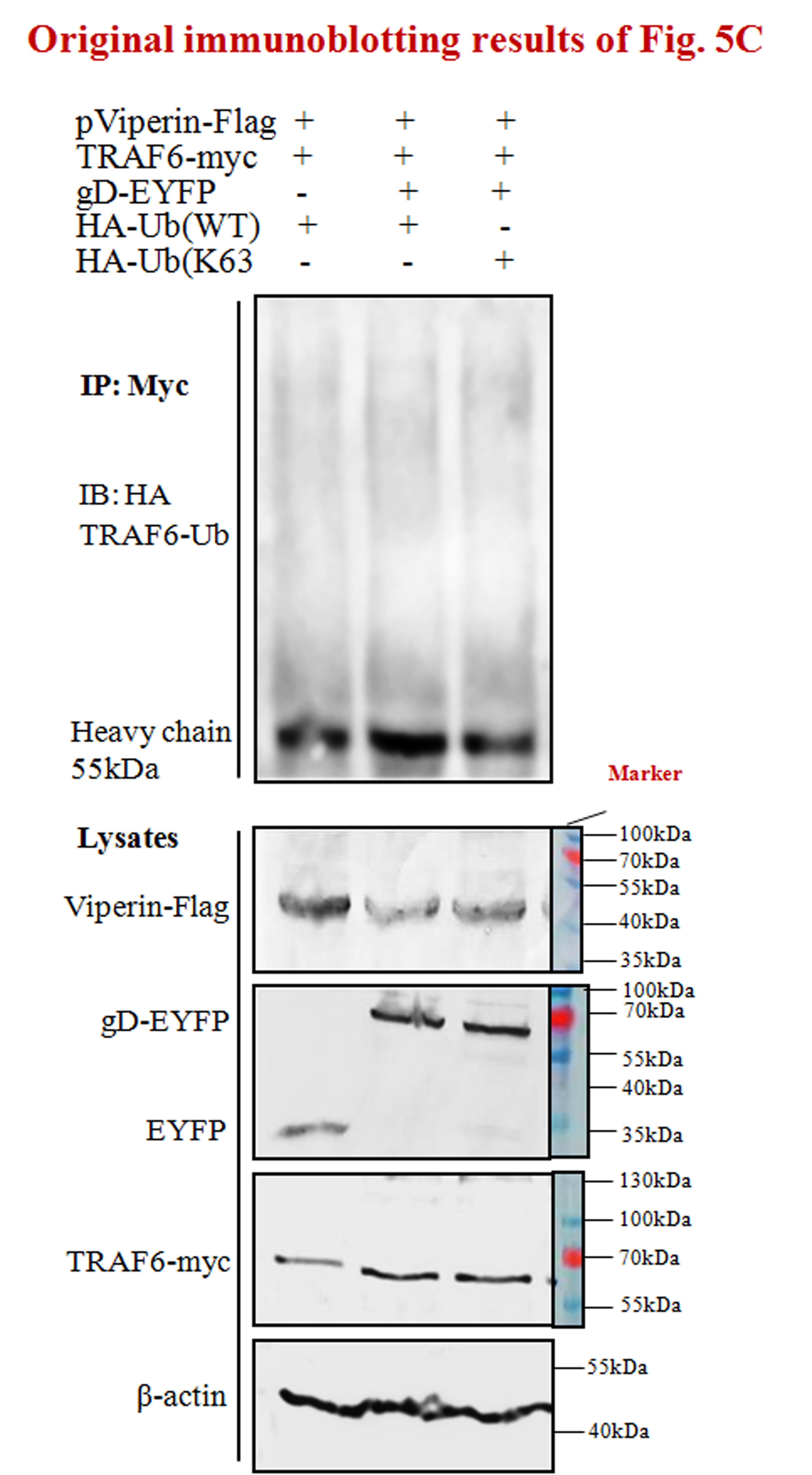

Supplement: Supplementary Material 4 — Original immunoblotting results of Figure 5C. [file Image_4.jpg]

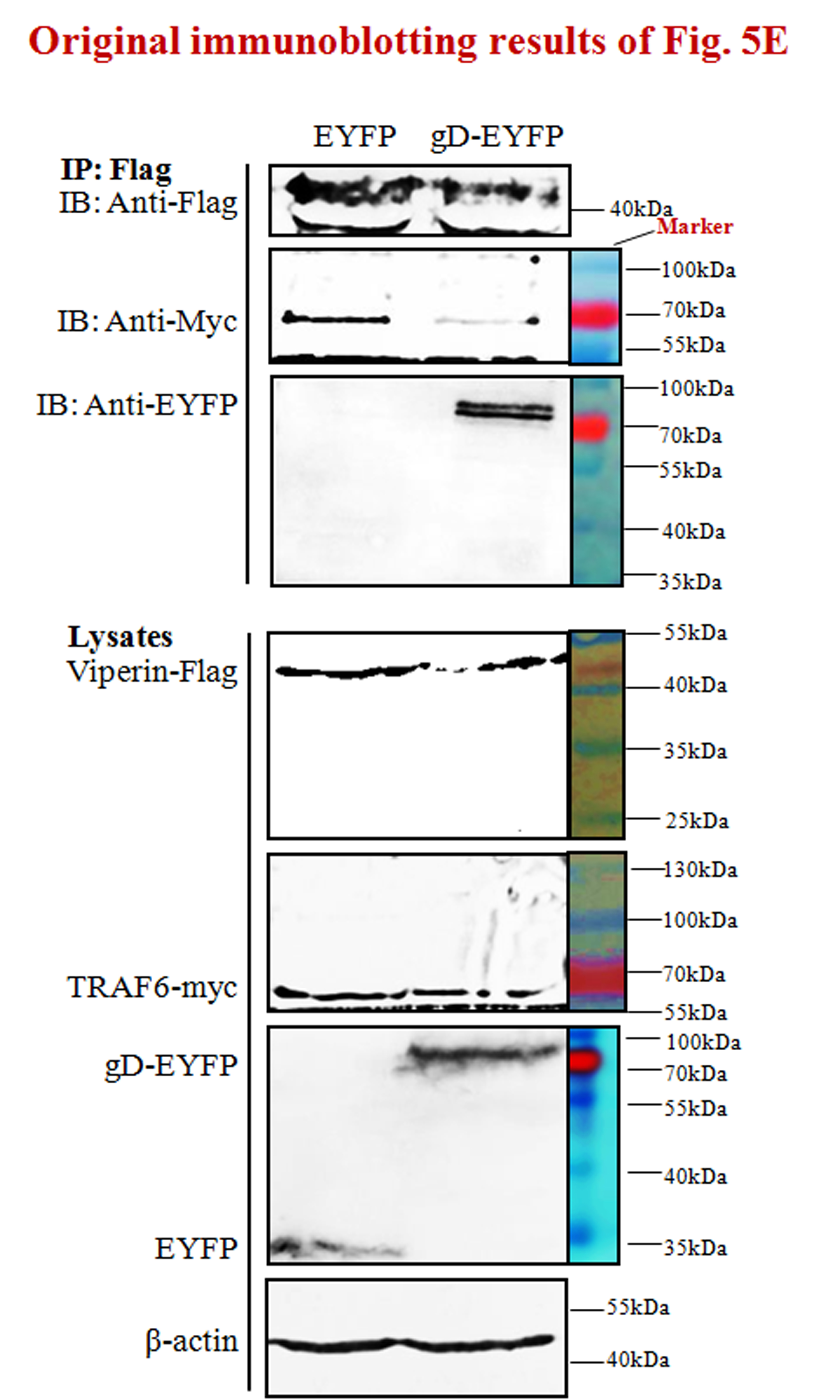

Supplement: Supplementary Material 5 — Original immunoblotting results of Figure 5E. [file Image_5.jpg]

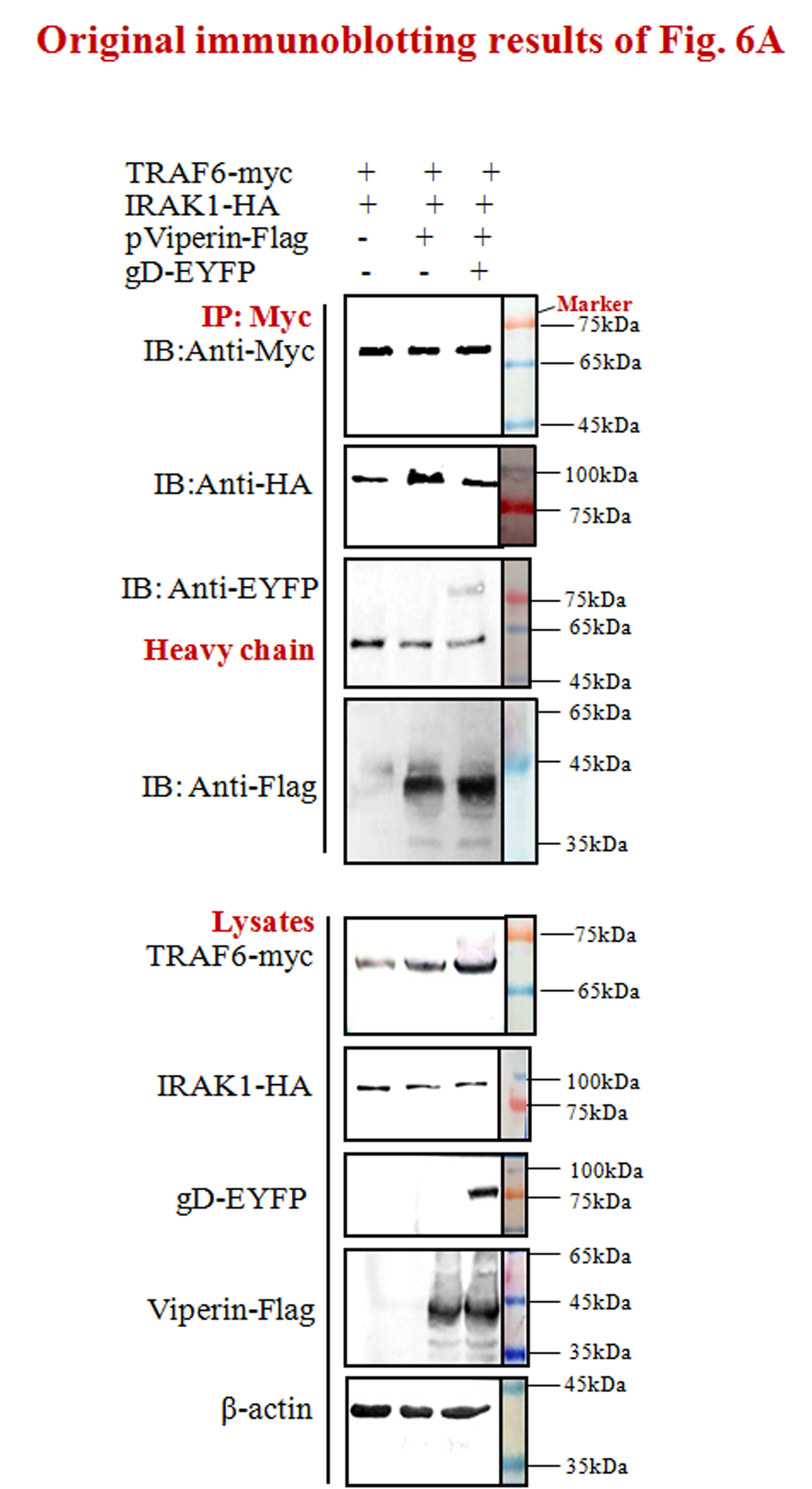

Supplement: Supplementary Material 6 — Original immunoblotting results of Figure 6A. [file Image_6.jpg]
